# Bacterial ubiquitin ligase engineered for small molecule and protein target identification

**DOI:** 10.1101/2025.03.20.644192

**Published:** 2025-03-22

**Authors:** James S. Ye, Abir Majumdar, Brenden C. Park, Miles H. Black, Ting-Sung Hsieh, Adam Osinski, Kelly A. Servage, Kartik Kulkarni, Jacinth Naidoo, Neal M. Alto, Margaret M. Stratton, Dominique Alfandari, Joseph M. Ready, Krzysztof Pawłowski, Diana R. Tomchick, Vincent S. Tagliabracci

**Affiliations:** 1 Department of Molecular Biology, University of Texas Southwestern Medical Center, Dallas, TX 75390, USA.; 2 Howard Hughes Medical Institute, University of Texas Southwestern Medical Center, Dallas, Texas 75390, USA.; 3 Department of Internal Medicine, University of Texas Southwestern Medical Center, Dallas, TX 75390, USA.; 4 Department of Biochemistry, University of Texas Southwestern Medical Center, Dallas, TX 75390, USA.; 5 Department of Microbiology, University of Texas Southwestern Medical Center, Dallas, TX 75390, USA.; 6 Department of Biochemistry and Molecular Biology, University of Massachusetts Amherst, Amherst, MA 01003, USA.; 7 Department of Veterinary and Animal Sciences, University of Massachusetts Amherst, Amherst, MA 01003, USA.; 8 Department of Biophysics, University of Texas Southwestern Medical Center, Dallas, TX 75390, USA.; 9 Harold C. Simmons Comprehensive Cancer Center, University of Texas Southwestern Medical Center, Dallas, Texas 75390, USA.; 10 Hamon Center for Regenerative Science and Medicine, University of Texas Southwestern Medical Center, Dallas, Texas 75390, USA.

## Abstract

The *Legionella* SidE effectors ubiquitinate host proteins independently of the canonical E1-E2 cascade. Here we engineer the SidE ligases to develop a modular proximity ligation approach for the identification of targets of small molecules and proteins, which we call SidBait. We validate the method with known small molecule-protein interactions and use it to identify CaMKII as an off-target interactor of the breast cancer drug ribociclib. Structural analysis and activity assays confirm that ribociclib binds the CaMKII active site and inhibits its activity. We further customize SidBait to identify protein-protein interactions, including substrates for enzymes, and discover the F-actin capping protein (CapZ) as a target of the *Legionella* effector RavB during infection. Structural and biochemical studies indicate that RavB allosterically binds CapZ and decaps actin, thus functionally mimicking eukaryotic CapZ interacting proteins. Collectively, our results establish SidBait as a reliable tool for identifying targets of small molecules and proteins.

*Legionella pneumophila* is an intracellular bacterial pathogen that translocates over 330 effector proteins that disrupt essential cellular processes^1^, including ubiquitination^2–4^ and cytoskeleton remodeling^5^, to create a specialized replicative niche called the *Legionella*-containing vacuole (LCV). The SidE family of effectors (SdeA, SdeB, SdeC and SidE; hereafter referred to collectively as “SidE”) catalyze the ligation of ubiquitin (Ub) to Ser and Tyr residues on host proteins independently of the cellular E1 and E2 Ub-conjugating enzymes^4,6,7^. The SidE effectors, which are spatially and temporally regulated during *Legionella* infection^8–13^, ubiquitinate several host proteins to protect the LCV from degradation by the host^14^. This process involves the SidE ADP-ribosyltransferase (ART) domain, which transfers ADP-ribose from NAD^+^ to Arg42 on Ub. Subsequently, the SidE phosphodiesterase (PDE) domain hydrolyzes the phosphoanhydride bond in ADP-ribose and thereby attaches Ub to Ser or Tyr residues on host proteins through a phosphoribose (pR) linkage (Fig. 1a, b). Phosphoribosyl ubiquitination of proteins by the SidE ligases does not use the Ub C-terminal diglycine motif, nor does it require any Lys residues present in Ub or SidE substrates^4^. This orthogonality to conventional ubiquitination in the mammalian cell led us to envision a chemical proteomic approach for target identification by means of SidE-catalyzed protein capture.

Common strategies for target identification include diazirine photochemistry to crosslink ligands to proteins^15^, engineered enzyme fusions that generate diffusible reactive species^16–18^, modified ubiquitin-like proteins^19,20^ and others^21^. Each approach has drawbacks that present challenges in target validation; thus, the pursuit of complementary methods to uncover the mechanisms of action of small molecules and the substrates of enzymes remains a major objective. Here we develop a modular proximity ligation method, called SidBait, that utilizes the SidE ligase to covalently capture proximal interacting targets of a small molecule or a protein of interest. We showcase SidBait’s utility by identifying the known targets of small molecules and proteins, thereby confirming its potential for precise target identification. We use SidBait to discover an off-target of the cyclin-dependent kinase 4/6 (CDK4/6) inhibitor ribociclib and elucidate the function of the uncharacterized *Legionella* effector RavB.

## Results

### Development of SidBait for the identification of targets of small molecules.

The SidE ligases do not require a consensus sequence surrounding the Ser/Tyr residue for phosphoribosyl ubiquitination of substrates, resulting in low substrate specificity in vitro and when overexpressed in cells^3,22^. While the ART domain specifically ADP-ribosylates Arg42 of Ub, the PDE domain is relatively indiscriminate in its substrate preferences. Importantly, the ART and PDE activities of SidE can be decoupled; for example, ADP-ribosylated Ub can be used as a substrate for the isolated PDE domain in the absence of the ART domain^8^.

**Figure 1:**
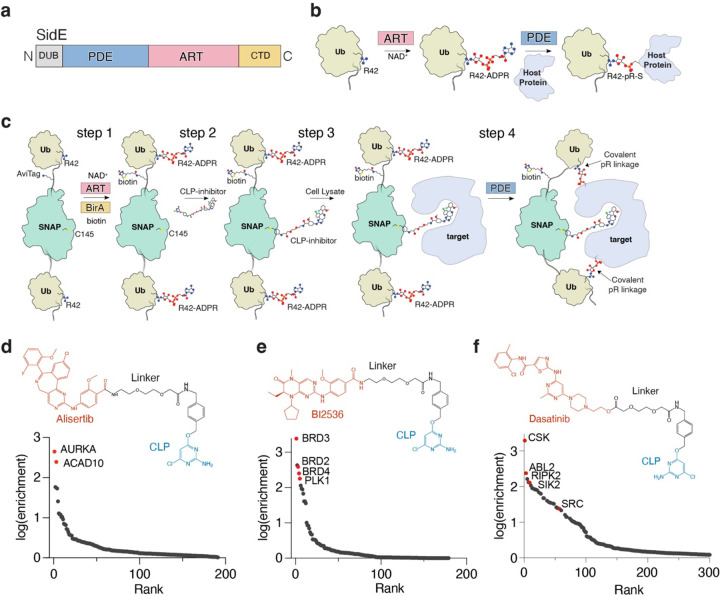
The SidBait approach to discover protein targets of small molecules. **(a)** Domain architecture of the SidE ligases depicting the phosphodiesterase (PDE; light blue) domain and ADP-ribosyltransferase domain (ART; pink). The deubiquitinase (DUB) and C-terminal domain (CTD) are also shown. **(b)** The enzymatic reaction catalyzed by the SidE ligases. In the first step of the reaction, the ART domain ADP-ribosylates Arg^42^ of Ub using NAD^+^ as a co-substrate. Subsequently, the PDE domain hydrolyzes the phosphodiester bond and attaches the Ub to Ser and Tyr residues on host proteins. **(c)** Overall schematic of the SidBait technology. In **step 1**, a 6xHis-Ub-Avi-SNAP-Ub fusion protein is ADP-ribosylated by the SidE ART domain and biotinylated by BirA. In **step 2**, a chloropyrimidine (CLP) derivative of a small molecule is conjugated to the SNAP tag. In **step 3**, the probe is incubated with a cell lysate and the small molecule engages its target. In **step 4**, the SidE PDE domain is then added to covalently crosslink proximal interacting proteins through a phosphoribose (pR) linkage to the bait. Interacting proteins are then affinity purified by avidin pulldown and subjected to LC-MS/MS for identification. **(d-f)** Plots of fold enrichment of proteins from SidBait-alisertib **(d)**, SidBait-BI2536 **(e)**, and SidBait-dasatinib **(f)** experiments over pulldowns with the SidBait^C145A^ control, which cannot accommodate the CLP probe. Known direct interactors are colored in red. Fold enrichment values are averaged from three independent experiments. Chemical structures of the CLP-tagged alisertib **(d)**, BI2536 **(e)** and dasatinib **(f)** derivatives are shown above the plots.

We envisioned a strategy to exploit the nonspecificity of the SidE effectors to identify proximal interacting proteins of small molecules. We generated a bacterial expression construct containing a 6xHis tag to aid in purification, an avidin-tag for target enrichment and a SNAP-tag, which can covalently conjugate a small molecule containing a chloropyrimidine (CLP) moiety^23^, flanked by a pair of Ubs (Fig. 1c). We also mutated the diglycine motif in Ub to dialanine to prevent recognition by the endogenous eukaryotic ubiquitination machinery. We co-expressed the fusion protein with the untagged ART domain of SidE (SdeA^519−1100^) and the biotin ligase BirA in *E. coli* and purified the fusion protein by Ni-NTA affinity chromatography (Fig. 1c; step 1). Intact mass spectrometry analysis revealed full incorporation of 2 ADPR molecules and 1 biotin (Supplementary Fig. 1a). The fully modified protein is hereafter referred to as SidBait.

We incubated SidBait with SidE and observed NAD^+^-independent autoubiquitination, indicated by the high molecular weight crosslinks, thus confirming that the ADP-ribosylated Ubs serve as substrates for the SidE PDE domain (Supplementary Fig. 1b). To attach a small molecule to the bait, we synthesized a CLP derivative of the Aurora kinase A (AURKA) inhibitor alisertib (Fig. 1d; top), which inhibits AURKA with a similar IC_50_ to the unmodified drug^24,25^. We then incubated CLP-alisertib with SidBait and observed full occupancy of the compound into the SNAP-tag (SidBait-alisertib), but not a SNAP^C145A^ mutant, which is incapable of incorporating the CLP tag (Fig. 1c; step 2, Supplementary Fig. 1c, d).

To determine whether SidBait-alisertib could enrich AURKA, we incubated the probe with a HEK293 cell lysate (Fig. 1c; step 3), added the SidE PDE domain (SdeA^178−1100^) to crosslink any proximal interacting proteins (Fig. 1c; step 4) and enriched the bait by avidin pulldown. As a control, we used SidBait containing the SNAP^C145A^ mutant. Mass spectrometry analysis identified AURKA and the known off-target alisertib interactor ACAD10^26^ as the most highly enriched proteins (Fig. 1d, Supplementary Table 1). Free alisertib outcompeted SidBait-alisertib for AURKA, confirming that the target binding was solely due to the SidBait-alisertib probe (Supplementary Fig. 1e). These experiments also revealed that AURKA was completely depleted from the cell lysate by the probe.

As further validation for this approach, we synthesized CLP derivatives of the dual PLK1 and BRD4 inhibitor BI2536^27^, and the SRC-family tyrosine kinase inhibitor dasatinib^28^ (Fig. 1e, f; top), and performed SidBait experiments. Mass spectrometry analysis of the enriched baits identified the expected targets of these drugs (Fig. 1e, f, Supplementary Table 2, 3). Thus, SidBait identifies protein targets of small molecules.

### SidBait identifies CaMKII as a target of ribociclib.

**Figure 2:**
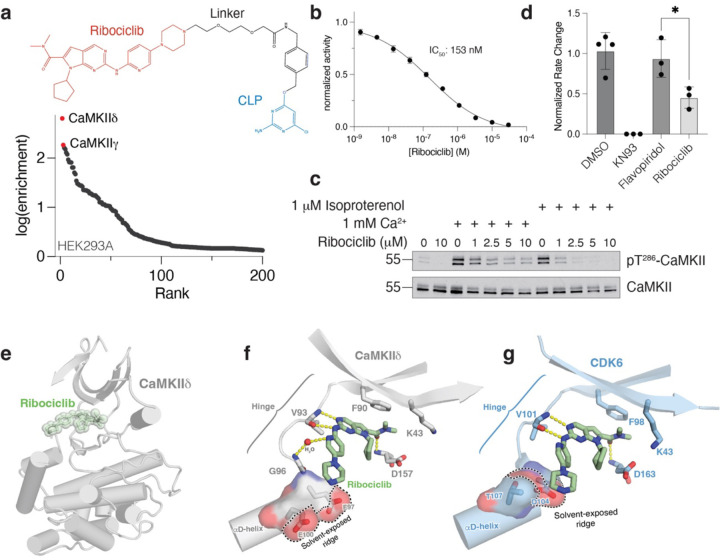
SidBait identifies ribociclib as an active site inhibitor of CaMKII. **(a)** Plot of fold enrichment of proteins from SidBait-ribociclib experiments in HEK293A cells over the SidBait^C145A^ control. Fold enrichment values are averaged from three independent experiments. The chemical structures of CLP-tagged ribociclib is shown above the plot. **(b)** In vitro CaMKIIδ activity assay in the presence of varying concentrations of ribociclib. The IC_50_ of ribociclib is shown in the inset. Error bars represent the S.E.M. of three replicates. **(c)** Protein immunoblotting of human cardiomyocyte lysates that have been stimulated with Ca^2+^ or isoproterenol in the presence of varying concentrations of ribociclib. Total CaMKII and pThr^286^ CaMKII are shown as an indication of CaMKII activity. **(d)** Fold change in beating rate of cardiomyocytes following treatment with the CaMKII inhibitor KN93, the pan-CDK inhibitor flavopiridol or the CDK4/6 inhibitor ribociclib. Results represent the mean of three independent experiments. **(e)** Overall structure of the CaMKIIδ kinase domain (grey) with ribociclib (green) bound in the active site. **(f, g)** Zoomed in view of the active sites of CaMKII in grey **(f)** and CDK6 in light blue **(g; PDB: 5L2T)** depicting the interactions between the kinase domain and ribociclib (green).

We next performed SidBait using the clinically approved CDK4/6 inhibitor ribociclib, which is used in the treatment of breast cancer^29^. Although we initially failed to detect CDK4/6, we unexpectedly identified calcium/calmodulin-dependent protein kinase type II (CaMKII) paralogs as top hits in HEK293, human cardiomyocyte, and MCF7 cell lysates (Fig. 2a, Supplementary Fig. 2a, b, Supplementary Table 4). However, incubation of SidBait-ribociclib in a cell lysate for 72 hours prior to the addition of the SidE PDE domain resulted in the enrichment of CDK4 (Supplementary Fig. 2c, Supplementary Table 4), demonstrating that incubation time prior to crosslinking is a variable that can be strategically optimized.

Consistent with the SidBait experiments, ribociclib inhibited CaMKIId activity in vitro and in cultured cardiomyocytes (Fig. 2b, c). Moreover, ribociclib-treated cardiomyocytes showed a significant decrease in beating rate, a known effect of CaMKII inhibition^30^ (Fig. 2d). We determined the crystal structure of the kinase domain of CaMKIIδ (CaMKIIδ^11−309^; hereafter referred to as CaMKIIδ^kd^) bound to ribociclib (Fig. 2e, Supplementary Fig. 2d, Supplementary Table 1). Ribociclib occupies the active site of CaMKII in a manner analogous to CDK6^31^, with the dimethylcarboxamide group of ribociclib packing against the gatekeeper Phe90 (Fig. 2f, g). There are hydrogen bonding interactions within the CaMKIIδ^kd^ hinge region and with the backbone nitrogen of Asp157. Residues Glu97 and Glu100, which are on the αD-helix of CaMKII and coordinate substrates and the ribose hydroxyls of ATP^32^ (Fig. 2f), provide a solvent-exposed ridge, like the one in CDK6 (Asp104 and Thr107), to stabilize the piperazine ring of ribociclib^31^ (Fig. 2g). The lower potency of ribociclib for CaMKII compared to CDK4/6 is likely to arise from the presence of a leucine (CaMKII Leu92) in place of the histidine residue (CDK6 His100) that imparts selectivity for ribociclib^33^ (Fig. 2b, Supplementary Fig. 2e–g). Collectively, our structural analysis shows that CaMKIIδ readily accommodates ribociclib and may facilitate the development of more selective CaMKII and CDK4/6 inhibitors.

### Development of SidBait for the identification of protein targets

**Figure 3:**
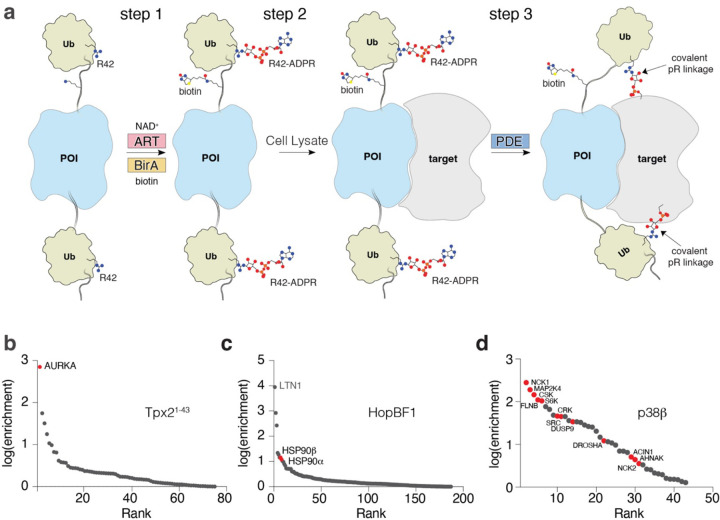
The SidBait approach to discover targets of proteins of interest. **(a)** Overall schematic of the SidBait approach with a protein of interest (POI). In **step 1**, a SidBait molecule in which the SNAP-tag is replaced with a POI is ADP-ribosylated by the SidE ART domain and biotinylated by BirA. In **step 2**, the probe is incubated with a cell lysate and the POI engages its binding partners. In **step 3**, the SidE PDE domain is then added to covalently crosslink proximal interacting proteins through a pR linkage to the bait. Interacting proteins are then affinity purified by avidin pulldown and subjected to LC-MS/MS for identification. **(b-d)** Plots of fold enrichment of proteins from SidBait-Tpx2^1−43^ **(b)**, SidBait-HopBF1 **(c)** and SidBait-p38β **(d)**. Fold enrichment values are averaged over three independent experiments. Known interacting partners are colored in red.

Replacing the SNAP-tag within SidBait with a protein of interest could, in principle, also be used to identify protein-protein interactions. To explore the modularity of SidBait, we replaced the SNAP-tag with: the Aurora A-activating segment of Tpx2 (Tpx2^1−43^); HopBF1, a bacterial effector kinase that phosphorylates eukaryotic HSP90^34^; and p38β, a member of the mitogen activated protein kinase (MAPK) family (MAPK11). We co-expressed the fusion proteins with the SidE ART domain and BirA in *E. coli* (Fig. 3a; step 1). Intact mass analysis revealed full incorporation of 2 ADPR molecules and 1 biotin (Supplementary Fig. 3a, b). We incubated these proteins with SidE and observed NAD^+^-independent autoubiquitination, thus confirming that the ADP-ribosylated Ubs serve as substrates for the SidE PDE domain (Supplementary Fig. 3c). We then incubated SidBait-Tpx2^1−43^, SidBait-HopBF1 and SidBait-p38β with HEK293 cell lysates (Fig. 3a; step 2), added the SidE PDE domain to crosslink any proximal interacting proteins (Fig. 3a; step 3) and enriched the baits by avidin pulldown. Mass spectrometry analysis identified AURKA as highly enriched in the SidBait-Tpx2^1−43^ experiment (Fig. 3b, Supplementary Table 5), and HSP90 in the SidBait-HopBF1 experiment (Fig. 3c, Supplementary Table 6). SidBait-p38β identified several interactors of p38β, including direct substrates and the upstream kinase MAP2K4^35^ (Fig. 3d, Supplementary Table 7). Thus, SidBait identifies protein-protein interactions, including substrates of enzymes and enzymes for substrates.

### The *Legionella* effector RavB targets the F-actin capping complex CapZ

**Figure 4:**
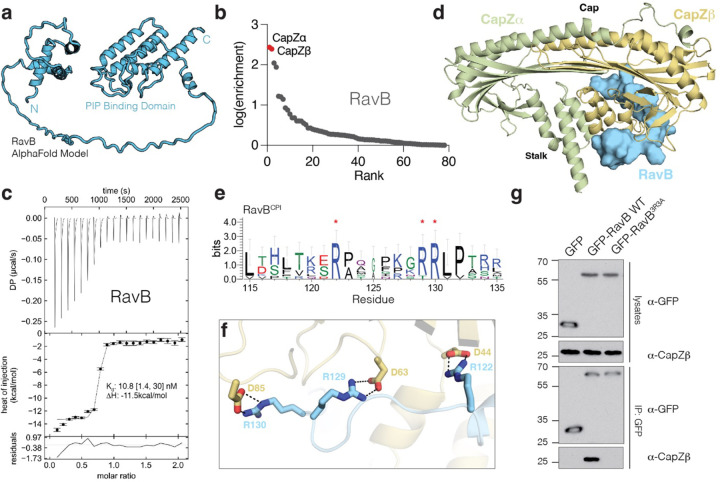
SidBait identifies CapZ as a binding partner of the *Legionella* effector RavB. **(a)** Structural model of RavB predicted by AlphaFold2. **(b)** Plot of fold enrichment of proteins from SidBait-RavB. Fold enrichment values are averaged over three independent experiments. **(c)** Isothermal titration calorimetry (ITC) trace showing the binding of RavB (injected) to CapZ (cell). *K*_d_ and enthalpy values are shown in the inset with a 68.3% confidence interval. **(d)** Overall structure of RavB (cyan) bound to CapZα (green) and CapZβ (yellow). The F-actin binding region (“Cap”) and the RavB binding region (“Stalk”) are labeled. **(e)** Sequence logo representing conservation of the RavB^CPI^ compiled across 45 homologs. Conserved arginine residues that directly participate in electrostatic interactions with CapZ are annotated with red asterisks. **(f)** Zoomed in view depicting the direct electrostatic interactions between RavB^CPI^ (cyan) and CapZβ stalk (yellow). **(g)** Protein immunoblotting of lysates and anti-GFP-immunoprecipitates from HEK293 cells that have been transfected with GFP, GFP-RavB or the GFP-RavB^3R3A^. anti-GFP and anti-CapZβ immunoblots are shown.

The identification of host targets of bacterial effectors is a significant challenge in host-pathogen biology. To test whether SidBait can be applied to these questions, we performed SidBait with the uncharacterized *Legionella* effector RavB (lpg0030). Analysis of the AlphaFold structural prediction suggests that RavB contains a helical N-terminal segment and a lipid binding C-terminal domain connected by a long unstructured linker (Fig. 4a)^36^. SidBait-RavB identified both subunits (CapZα and CapZβ) of the heterodimeric F-actin capping complex (CapZ) (Fig. 4b, Supplementary Table 8) as the target of RavB. Free RavB outcompeted SidBait-RavB for CapZ, confirming that the target binding was due solely to the presence of RavB (Supplementary Fig. 4a, b). CapZ binds to the barbed ends of actin filaments (F-actin) and regulates actin dynamics and the phagocytosis of bacteria^37^. RavB and CapZ formed a stable heterotrimeric complex with a *K*_d_ of 10.8 nM (Fig. 4c).

We solved the structure of the RavB:CapZ complex to a resolution of 2.00 Å. The structure resolved CapZ bound to an internal segment of RavB^115−139^ (Fig. 4d, Supplementary Table 1). CapZ consists of a heterodimer of CapZα and CapZβ, which produce a mushroom shaped structure with an α-helical stalk and an F-actin binding cap. RavB^115−139^ wraps around the stalk of the CapZ mushroom distal to the F-actin binding cap. RavB^115−139^ contains a highly conserved motif, ^115^L(X)_6_R(X)_6_RRLP^132^ (Fig. 4e) (hereafter referred to as RavB Capping protein interacting motif; RavB^CPI^), in which the arginines form electrostatic interactions with Asp44, Asp63, and Asp85 of CapZβ (Fig. 4f). Alanine substitutions of Arg122, Arg129 and Arg130 (RavB^3R3A^) abolished RavB binding to CapZ in vitro and in cells (Fig. 4g, Supplementary Fig. 4c). Likewise, a synthesized RavB^108−148^ peptide was able to bind CapZ with high affinity (Supplementary Fig. 4d). Thus, RavB^CPI^ is necessary and sufficient for its interaction with CapZ.

**Figure 5:**
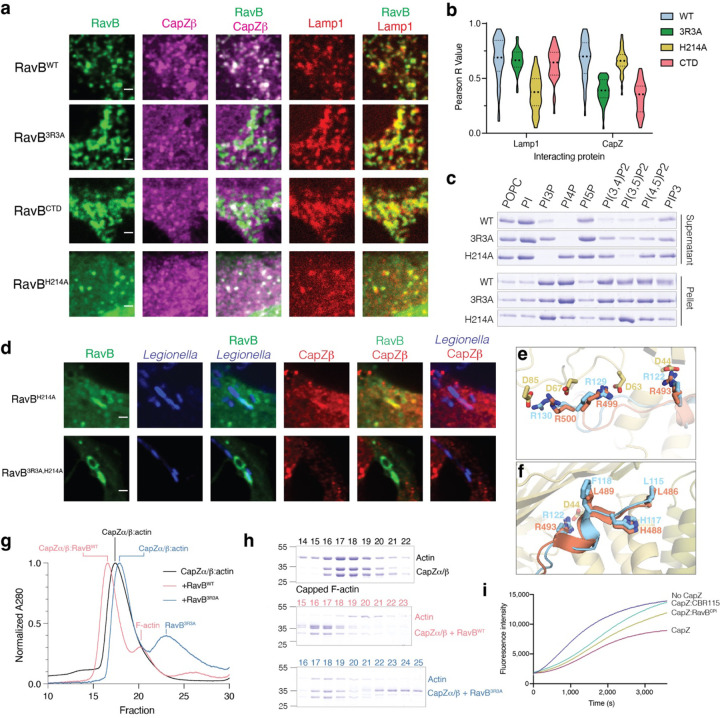
RavB acts as a lipid bound F-actin decapping protein. **(a)** Immunofluorescence microscopy of HeLa cells expressing EGFP-RavB^WT^ or various mutants, mTagBFP-Lamp1 and endogenous CapZβ. Scale bar represents 2 μm. **(b)** Plot depicting Pearson R Value of colocalization between transfected GFP-RavB, or various RavB mutants with mTagBFP-Lamp1 and endogenous CapZβ. Each comparison was calculated using 40 cells across 3 independent experiments. **(c)** Liposome sedimentation assay with different phospholipids and RavB or mutants. RavB bound liposomes were separated into supernatant (unbound) or pellet (bound) by high-speed centrifugation and RavB was visualized by Coomassie staining. **(d)** Immunofluorescence microscopy of mCherry-*Legionella*-infected (pseudocolored in blue) COS-7 cells expressing EGFP-RavB^H214A^ or EGFP-RavB^3R3A/H214A^. Endogenous CapZβ was also visualized (red). Scale bar represents 2 μm. **(e, f)** Zoomed in view of CapZ (yellow):RavB (cyan) structure with human CD2AP (orange, PDB: 3LK4) overlayed depicting direct electrostatic interactions **(e)** or the N-terminal segment of the RavB and CD2AP CPIs **(f)**. **(g)** Size exclusion chromatography depicting the A_280_ traces from the actin decapping experiments. CapZ-capped F-actin is shown in black, purified CapZ-capped F-actin incubated with RavB^WT^ is in red, and CapZ with the RavB^3R3A^ mutant in green. **(h)** Proteins present in fractions from size exclusion runs in **(g)** were visualized by Coomassie staining. **(i)** Pyrene-actin polymerization assays demonstrating that the RavB^CPI^ decaps actin. The polymerization of actin was measured in the presence of a buffer control (blue), CapZ (maroon), CapZ and the known decapping peptide CBR115 (teal), and CapZ and the Rav^CPI^ peptide (green). Assays were run with 2 μM G-actin, 50 nM CapZ and 1.25 μM decapping peptide.

RavB also contains a C-terminal phosphatidylinositol phosphate (PIP) binding domain (RavB^CTD^)^38^ (Fig. 4a). In HeLa cells, EGFP-tagged RavB recruited CapZ to LAMP-1 positive vesicular structures, suggesting ectopically expressed RavB exhibits lysosomal localization (Fig. 5a, b, Supplementary Fig. 5a, b). RavB^3R3A^ and RavB^CTD^ also localized to the lysosome, but were unable to recruit CapZ. RavB and RavB^3R3A^ bound PI3P, PI4P, and PI(3,5)P_2_, while the H214A mutant––predicted to disrupt PIP binding––was markedly impaired (Fig. 5c, Supplementary Fig. 5c). Likewise, RavB^H214A^ failed to localize to the lysosome when expressed in cells (Fig. 5a, b, Supplementary Fig. 5a, b). In *Legionella* infected cells, GFP-RavB^H214A^ but not RavB^3R3A/H214A^ co-localized with CapZ on the LCV (Fig. 5d).

The CapZ interacting proteins, CD2AP, CARMIL1 and CKIP-1, decap actin and contain membrane targeting domains used to spatially regulate actin dynamics^37^. These proteins also bind in the same allosteric groove as RavB using a similar CPI motif^39^ (Fig. 5e, f). Indeed, RavB, but not RavB^3R3A^, displaced CapZ from F-actin (Fig. 5g, h). Furthermore, RavB^CPI^ and the CapZ binding region from CARMILI (CBR115)^40^ decapped actin and promoted its polymerization (Fig. 5i, Supplementary Fig. 5d). Collectively, our results suggest that RavB functionally mimics eukaryotic CapZ interacting proteins by allosterically binding CapZ and decapping F-actin.

### Expanding the applications of SidBait

**Figure 6:**
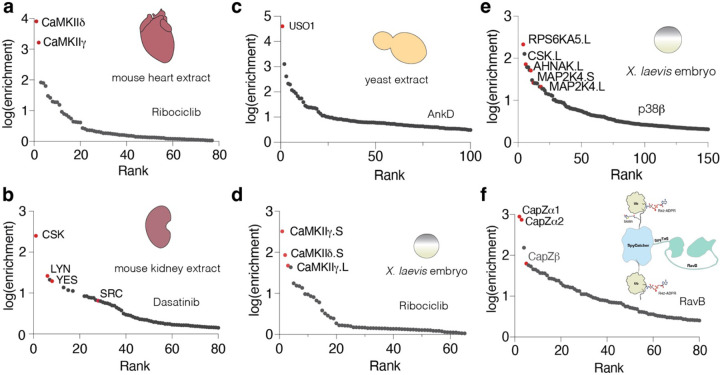
Additional applications of SidBait. **(a, b)** Plots of fold enrichment of proteins from SidBait-ribociclib in a mouse heart extract **(a)** and SidBait-dasatinib from a mouse kidney extract **(b)** over pulldowns with the SidBait^C145A^ control. **(c)** Plot of fold enrichment of proteins from SidBait-AnkD from yeast extracts over pulldowns with the SidBait protein control. **(d, e)** Plots of fold enrichment of proteins from SidBait-ribociclib **(d)** and SidBait-p38b **(e)** from live *X. laevis* embryos over pulldowns with the respective controls. **(f)** Plot of fold enrichment of proteins from a SpyTag/SpyCatcher fusion of RavB over pulldowns with the non-conjugated control. Known interactors are shown in red.

A key advantage of SidBait is that the technique is agnostic to the source of target proteins. Using baits we had already tested against cell lysates, we expanded our protein sources to include animal tissues and yeast. We performed SidBait experiments using ribociclib and dasatinib in a homogenized mouse heart and kidney, respectively. As expected, SidBait-ribociclib resulted in an enrichment of CaMKIIδ and γ (Fig. 6a, Supplementary Table 4), while SidBait-dasatinib yielded the tyrosine kinases Csk, Lyn, Yes and Src (Fig. 6b, Supplementary Table 3). From yeast extracts, SidBait experiments using the bacterial effector AnkD resulted in the enrichment of its expected target USO1^41^ (Fig. 6c, Supplementary Table 9).

To evaluate the *in vivo* applicability of SidBait, we serially injected various baits and the SidE PDE protein into live *X. laevis* embryos. Using SidBait-ribociclib, SidBait-p38β, and SidBait-dasatinib, we successfully captured many of the expected targets (Fig. 6d, e Supplementary Fig. 6a, Supplementary Tables 3,4,7), thus establishing the method’s functionality in a live system.

A limitation of SidBait is that bait proteins must be expressed and ADP-ribosylated in *E. coli* because SidE-dependent ADP-ribosylation of endogenous ubiquitin in eukaryotic systems causes cytotoxicity^6^. To circumvent this issue, we used the SpyTag-SpyCatcher system^42^, which enables the fusion of two separate polypeptides via an isopeptide bond (Supplementary Fig. 6b). As a proof-of-concept, we purified the Ub-SpyCatcher-Ub and SpyTag-RavB proteins separately, conjugated them and performed a SidBait experiment from a cell lysate. As expected, the CapZ complex was highly enriched (Fig. 6f, Supplementary Table 8). We envision this approach to be particularly useful for applications that require alternative expression systems for bait proteins.

## Discussion

In this work we introduce SidBait, a proximity ligation approach that complements current methodologies to identify targets of small molecules and proteins. We validate the method by recapitulating known bait-target pairs and demonstrate its utility as a hypothesis-generator by identifying and further substantiating previously unexplored interactions. These discoveries were facilitated by key features of SidBait that distinguish it from existing proximity ligation techniques: modularity, which allows for efficient and reliable target identification of both small molecules and proteins using the same method; a covalent linkage between the bait and target, which permits stringent washing to reduce background and false positives; and widely tunable experimental parameters, such as temperature (4–37 °C), incubation time and concentrations of bait and lysate.

For example, our initial failure to detect CDK4 with ribociclib (Fig. 2a) was likely due to the inability of CDK4 to bind ATP-competitive inhibitors in its heterotrimeric CDK4:CyclinD:p27 form^43^. This led us to attempt a significantly longer incubation with SidBait-ribociclib than proximity ligation approaches typically permit, thus allowing more time to capture the monomeric CDK4 that exists in equilibrium (Supplementary Fig. 2c). Meanwhile, our unexpected results showing ribociclib to be a potent CaMKII inhibitor are consistent with other reports that have noted this interaction in passing^31,44^. Interestingly, some breast cancer patients develop serious cardiovascular side effects upon treatment with ribociclib, including long QT syndrome^45^. Because CaMKII responds to calcium flux in cardiomyocytes and regulates cardiac pacemaking^46^, we speculate that ribociclib targeting CaMKII may contribute to the observed heart toxicity in a small subset of patients^47^.

While SidBait is effective when injected into frog embryos (Fig. 6d, e), its ability to be used in lysates, particularly for small molecule target identification, is a notable strength of the technology. For instance, our results with SidBait-alisertib using HEK293 cell lysates unambiguously identified the intended target, Aurora A, as well as ACAD10, an off-target interactor^26,48^, as the two most highly enriched targets. In contrast, genetically encoded proximity ligation methods that also used alisertib from HEK293 cells failed to identify ACAD10^18^, which is a mitochondrial protein^49^; genetically encoded systems are unlikely to penetrate the mitochondria unless programmed to do so. Thus, SidBait is not limited to any specific subcellular compartment as it effectively interacts with all proteins within the lysate—a particularly valuable feature when identifying off-targets of small molecules.

In two other small molecule target identification studies performed in cells with dasatinib, the authors report interactors using photocatalysis-mediated crosslinking^50^ and proximity-based compound-binding protein identification^51^. Unlike these methods, we successfully identified SIK2, a known dasatinib interactor^52,53^ that is not a Src-family kinase.

Several pieces of evidence suggest that SidBait can capture transient interactions. Our data with p38β demonstrates that we can enrich the upstream kinase, MAP2K4, which interacts with p38β in the high micromolar range^35^ (Fig. 3d). Furthermore, we can enrich p38β substrates, including several that, to the best of our knowledge, do not form stable complexes with p38β. Finally, the HSP90 kinase HopBF1 does not stably interact with HSP90 in vitro and has a *K*_m_ of ~3 μM for HSP90^34^. Using SidBait-HopBF1, we successfully enriched HSP90 from HEK293 cells (Fig. 3c).

The unambiguous hits from the SidBait-RavB experiment allowed us to focus on the effector’s mechanism as an actin decapper. *Legionella* frequently exploits the host cytoskeletal network, with multiple effectors specifically targeting actin-regulating proteins or actin itself. Similarly to RavB, MavH binds CapZ and enhances actin polymerization at the LCV during phagocytosis, thus increasing bacterial uptake^5^. Likewise, LegK2 inhibits actin nucleation at the LCV to evade lysosomal fusion and bacterial destruction^54^. Because CapZ is required for vesicle trafficking to lysosomes^55^, we propose that RavB may be working in concert with other effectors located at the LCV to block actin- and CapZ-mediated lysosomal trafficking, thus ensuring the survival of the bacterium.

We have engineered the SidE all-in-one ubiquitin ligase to identify targets of small molecules and proteins. As with any other technique, we do not expect SidBait to work for every use case. However, we contend that between SidBait and the other proximity ligation techniques, there are overlapping but also mutually exclusive use cases. We anticipate that its versatility, orthogonal mechanism and ease of use may facilitate the discovery of important biomolecular interactions.

## Methods

### Cell lines

HEK293A were a gift from J. Jewell; COS-7 and MCF7 cells were from the ATCC; H9 cells were from WiCell, WA09 and HeLa cells were from ATCC.

### Antibodies

Aurora A D3E4Q Rb antibody (Cell Signaling, 14475S); Phospho-CaMKII D21E4 (Thr286) Rb antibody (Cell Signaling, 12716S); CaMKII (pan) D11A10 Rb antibody (Cell Signaling, 4436S); CAPZα1 Ms antibody (Proteintech, 66066–1-IG); CAPZβ F-actin-capping protein subunit beta Rb antibody (Sigma-Aldrich, AB6017); GAPDH Ms antibody (GA1R) (Abcam, ab125247); GFP Living Colors A.v. Ms antibody (JL-8) (Takara Bio, 632381); HSP90 α/β Ms antibody (F-8) (Santa Cruz, sc-13119); *Legionella pneumophila* LPS Ms antibody (ThermoFisher, AWB4CE4); Amersham ECL Rabbit IgG, HRP-linked whole Ab (from donkey) (Cytiva, NA934); Amersham ECL Mouse IgG, HRP-linked whole Ab (from sheep) (Cytiva, NXA931); Donkey anti-Rabbit IgG (H+L) Highly Cross-Adsorbed Secondary Antibody, Alexa Fluor 647 (ThermoFisher, A-31573)

### Small molecules

Alisertib (MedChem Express, HY-10971); Ribociclib (AdooQ BioScience, A13549 and Selleck Chemicals, S7440); KN-93 phosphate (Selleck Chemicals, S7423); Flavopiridol (Selleck Chemicals, S1230); CHIR99021 (Tocris, 4423); Wnt-C59 (Cayman Chemical, 16644); Isoproterenol (Sigma-Aldrich, I5752). CLP-ribociclib and CLP-dasatinib were synthesized by WuXi AppTec.

### Reagents

D-biotin (Goldbio, D-950–100); Streptavidin agarose resin (Goldbio, S-105–10); DMSO (Sigma, D2650–100ML); cOmplete, EDTA-free protease inhibitor (Sigma, 5056489001), DMEM (Fisher Scientific, 11–965-118); Fetal bovine serum (Sigma Aldrich, F-2442–500ML); Penicillin-streptomycin (Sigma Aldrich, P0781–100ML); PolyJet (SignaGen, SL100688); TransIT-X2 (mirusbio, MIR 6000); mTeSR Plus (Stemcell Technologies, 100–0276); Matrigel (Corning, 354277); RPMI 1640 (Thermo Fisher, 11875093); GFP-Catcher (Antibodies-Online, ABIN5311508); PIP strips (Cedarlane Labs, p-6001); Actin protein (Cytoskeleton, APHL99-C); Actin-pyrene polymerization kit (Cytoskeleton, BK003).

### Generation of Plasmids

The SidBait vector (6xHis-Avitag-Ub-MCS-Ub) was synthesized as a gBlock (IDT) and cloned into a pET-28a vector. The SNAPf and Tpx2^1−43^ constructs were synthesized as gBlocks (IDT) and the p38β construct was synthesized as a gene fragment (Twist Bioscience) and cloned into the SidBait vector. *E. americana* HopBF1 was PCR amplified from a pET-28a-based vector^34^ and cloned into the SidBait vector. Codon optimized SdeA (178–1100) was subcloned into ppSumo (a modified pET-28a based vector containing a 6X His tag and the yeast smt3 CDS) and used in SidBait experiments to crosslink baits to targets. Codon optimized SidE (SdeA^519−1100^) and *E. coli* BirA (amplified from BL21 gDNA) were cloned (untagged) into pET-Duet1 and used to ADP-ribosylate and biotinylate the SidBait proteins. RavB coding sequences were amplified by PCR using *Legionella pneumophila* genomic DNA (gDNA) as a template and cloned into pEGFP-C1, pProEx2, and ppSumo. Human CAMKIId^11−309^ was PCR amplified from cDNA and cloned into ppSumo. CBR115 (Glu964–Ser1078 of Human CARMIL1a)^40^ was synthesized as a gBlock (IDT) and cloned into ppSumo. FcγRIIB was PCR amplified from cDNA and cloned into a pcDNA vector containing a C-terminal Flag tag (pCCF).

All mutations were made using QuikChange site directed mutagenesis, and primers were designed with the online Agilent QuikChange Primer Design webpage.

### SidBait protein expression and purification

All pET-28a SidBait constructs (Ub-POI-Ub, Ub-SNAPf-Ub, or the Ub-MCS-Ub control) were co-transformed with pET-Duet1-BirA-SdeA^519−1100^ into Rosetta (DE3) *E. coli*. Cells were grown in 1 L LB in the presence of 50 μg ml^−1^ kanamycin, 100 μg ml^−1^ ampicillin and 1 mM biotin at 37 °C and induced at an OD_600_ of ~0.7 with 0.4 mM IPTG. Proteins were expressed overnight at 18 °C. Cultures were centrifuged at 3,000g for 10 minutes, and the bacterial pellets were resuspended in lysis buffer (50 mM Tris-HCl, pH 8.0, 300 mM NaCl, 25 mM imidazole, 1 mM DTT, 1 mM PMSF). Resuspended cells were lysed by sonication and the lysates were cleared by centrifugation at 30,000g for 30 minutes. The lysate was passed over Ni-NTA beads, which were washed with lysis buffer. The protein samples were eluted with elution buffer (50 mM Tris, pH 8.0, 300 mM NaCl, 300 mM imidazole, 1 mM DTT) and further purified by size-exclusion chromatography using a Superdex HiLoad S75 16/600 column (Cytiva) into 50 mM Tris, pH 8.0, 300 mM NaCl, 1 mM DTT. Complete ADP-ribosylation of ubiquitins and biotinylation of the AviTag was ensured by intact mass spectrometry analysis. Protein samples were flash frozen and stored at −80 °C until use.

### SidBait protein laddering assay

Five μg of SidBait protein were mixed with 5 μg SidE (SdeA^178−1100^) in 20 μl reactions in TBS-DTT buffer (50 mM Tris-HCl pH 8.0, 150 mM NaCl, 1 mM DTT) for 1 hr at room temperature. Reactions were terminated with 5X SDS-PAGE loading buffer (62.5 mM Tris-PO_4_ pH 6.8, 50% w/v glycerol, 6.25% SDS, 0.1% bromophenol blue, 5% 2-mercaptoethanol), separated by SDS-PAGE and visualized by Coomassie staining.

### SidE protein expression and purification

6xHis-ppSumo-SdeA^178−1100^ was transformed into Rosetta (DE3) *Escherichia coli*. Cells were grown in 1 L LB in the presence of 50 μg ml^−1^ kanamycin at 37 °C and protein expression was induced at an OD_600_ of ~0.7 with 0.4 mM IPTG. Protein was expressed overnight at 18°C. Cultures were centrifuged at 3,000g for 10 minutes, and the bacterial pellets were resuspended in lysis buffer (50 mM Tris-HCl, pH 8.0, 300 mM NaCl, 25 mM imidazole, 1 mM DTT, 1 mM PMSF). Resuspended cells were lysed by sonication and the lysates were cleared by centrifugation at 30,000g for 30 minutes. The lysate was passed over Ni-NTA beads, which were washed with lysis buffer. The Sumo-tagged SdeA^178−1100^ was eluted with elution buffer (50 mM Tris-HCl, pH 8.0, 300 mM NaCl, 300 mM imidazole, 1 mM DTT) and cleaved overnight at 4 °C with Ulp Sumo protease. The cleaved SdeA^178−1100^ was further purified by size-exclusion chromatography using a Superdex HiLoad S200 16/600 size exclusion column (Cytiva) into size exclusion buffer (50 mM Tris-HCl, pH 8.0, 150 mM NaCl, 1 mM TCEP). SdeA^178−1100^ was concentrated to 5 mg/mL, aliquoted, flash frozen and stored at −80 °C until use.

### RavB and CAMKIId expression and purification

6xHis-ppSumo-RavB and 6xHis-ppSumo-CAMKIId^11−309^ were transformed into Rosetta (DE3) *Escherichia coli*. Cells were grown in 1 L LB in the presence of 50 μg ml^−1^ kanamycin at 37 °C and protein expression was induced at an OD_600_ of ~0.7 with 0.4 mM IPTG. Proteins were expressed overnight at 18 °C. Cultures were centrifuged at 3,000g for 10 minutes, and the bacterial pellets were resuspended in lysis buffer (50 mM Tris-HCl, pH 8.0, 300 mM NaCl, 25 mM imidazole, 1 mM DTT, 1 mM PMSF). Resuspended cells were lysed by sonication and the lysates were cleared by centrifugation at 30,000g for 30 minutes. The lysate was passed over Ni-NTA beads, which were washed with lysis buffer. The protein samples were eluted with elution buffer (50 mM Tris-HCl, pH 8.0, 300 mM NaCl, 300 mM imidazole, 1 mM DTT) and cleaved overnight at 4 °C with Ulp Sumo protease. Cleaved proteins were further purified by size-exclusion chromatography using a Superdex 75 10/300 GL size exclusion column (GE) into size exclusion buffer (50 mM Tris-HCl, pH 8.0, 150 mM NaCl, 1 mM TCEP). Protein samples were flash frozen and stored at −80 °C until use.

### CBR115 expression and purification

6xHis-ppSumo-CBR115 was transformed into Rosetta (DE3) *Escherichia coli*. Cells were grown in 1 L LB in the presence of 50 μg ml^−1^ kanamycin at 37 °C and protein expression was induced at an OD_600_ of ~0.7 with 0.4 mM IPTG. Proteins were expressed overnight at 18 °C. Cultures were centrifuged at 3,000g for 10 minutes, and the bacterial pellets were resuspended in lysis buffer (50 mM Tris-HCl, pH 8.0, 300 mM NaCl, 25 mM imidazole, 1 mM DTT, 1 mM PMSF). Resuspended cells were lysed by sonication and the lysates were cleared by centrifugation at 30,000g for 30 minutes. The lysate was passed over Ni-NTA beads, which were washed with lysis buffer. The proteins were cleaved overnight at 4 °C with Ulp Sumo protease on the Ni-NTA beads. The supernatant was collected and were further purified by size-exclusion chromatography using a Superdex 75 10/300 GL size exclusion column (GE) into size exclusion buffer (50 mM Tris-HCl, pH 8.0, 150 mM NaCl, 1 mM DTT). Protein samples were flash frozen and stored at −80 °C until use.

### CapZ expression and purification

Chicken CapZα/β constructs (full length α/β for binding and polymerization assays; full length a and CapZb^1−244^ for crystallography experiments) in a pETDuet vector (a gift from Michael Rosen) were transformed into Rosetta (DE3) *Escherichia coli*. Cells were grown in 1 L LB in the presence of 100 μg ml^−1^ ampicillin at 37 °C and protein expression was induced at an OD_600_ of ~0.7 with 0.4 mM IPTG. Proteins were expressed overnight at 18 °C. Cultures were centrifuged at 3,000g for 10 minutes, and the bacterial pellets were resuspended in lysis buffer (50 mM Tris-HCl, pH 8.0, 300 mM NaCl, 25 mM imidazole, 1 mM DTT, 1 mM PMSF). Resuspended cells were lysed by sonication and the lysates were cleared by centrifugation at 30,000g for 30 minutes. The lysate was passed over Ni-NTA beads, which were washed with lysis buffer. The protein samples were eluted with elution buffer (50 mM Tris-HCl, pH 8.0, 300 mM NaCl, 300 mM imidazole, 1 mM DTT). CapZ was cleaved overnight at 4 °C with TEV protease. CapZ constructs were further purified by size-exclusion chromatography using a Superdex 200 HiLoad 16/600 size exclusion column (Cytiva) into size exclusion buffer (50 mM Tris-HCl, pH 8.0, 150 mM NaCl). Protein samples were concentrated to 10 mg/mL and flash frozen and stored at −80 °C until use.

### SidBait small molecule experiments

CLP-tagged small molecules were resuspended from powder in DMSO to a concentration of 10 mM. Preparation of SidBait-SNAPf^WT^-SidBait and SidBait-SNAPf^C145A^-SidBait probes were performed in parallel. Each SidBait-SNAPf-SidBait construct was diluted to 50 μM in 1x TBS, 1 mM TCEP and incubated with a 5x molar excess of the CLP-tagged small molecule at 37 °C for 30 minutes. Probes were desalted into 1x TBS, 1 mM TCEP to remove excess small molecule using a Zeba 7K MWCO spin desalting column (Thermo Fisher). Full incorporation of CLP-tagged molecules into the SidBait-SNAPf^WT^-SidBait probes, and lack of incorporation into the SidBait-SNAPf^C145A^-SidBait control, were ensured by intact mass spectrometry.

Confluent HEK293A cells in 15 cm dishes were washed twice with 10 mL of ice-cold PBS. After addition of 1.5 mL of 2x lysis buffer (100 mM Tris-HCl pH 7.5, 300 mM NaCl, 2 mM DTT, 2x Roche cOmplete protease inhibitor cocktail, 2 mM EDTA, 2% Triton X-100) per plate, cells were lysed with a cell scraper, aliquoted into 1.5 mL centrifuge tubes and cleared by centrifugation at 21,300g for 20 minutes. The supernatant was diluted to 5 mg/mL in 1x lysis buffer and used for subsequent experiments.

Cell lysates (300 μL) were added to SidBait probes such that the final probe concentration was 2–5 μM. Following incubation for 30–60 minutes, SdeA (1 μL from a 5 mg/mL stock) was added to the samples to initiate the crosslinking reaction and samples were incubated on ice for a further 2–12 h. Streptavidin-agarose beads (50 mL of 50% slurry) were added to the reactions and samples were nutated overnight at 4 °C. Beads were then spun at 800g for 1 minute at 4°C and washed successively with wash buffer 1 (2% SDS), wash buffer 2 (50 mM HEPES pH 7.5, 0.1% sodium deoxycholate, 1% Triton X-100, 1 mM EDTA, 500 mM NaCl), wash buffer 3 (10 mM Tris-HCl pH 8.0, 0.5% sodium deoxycholate, 0.5% NP-40, 1 mM EDTA, 250 mM LiCl) and wash buffer 4 (50 mM Tris-HCl pH 7.5) before mass spectrometry analysis.

### SidBait protein experiments

HEK293A lysates were prepared as described above. Cell lysates (300 μL) were added to SidBait proteins (30 μL of 30 μM protein) on ice. Following incubation for 30–60 minutes, SdeA (1 μL from a 5 mg/mL stock) was added to the samples to initiate the crosslinking reaction and samples were incubated on ice for an additional 2–12 hrs. Samples were bound to streptavidin-agarose beads and washed as described above before mass spectrometry analysis.

### SidBait competition assay

Lysates from HEK293 cells and SidBait probes were prepared as described above. Before addition of the probe to lysate, increasing concentrations of free-probe (native drug or untagged-protein) were added to the 300 μL aliquots of the lysate and mixed well by pipetting. The SidBait probe was then added to the lysates, incubated, and bound to beads as described above. The following day, before the first bead wash, the unbound lysate was collected. 40 μg of total unbound fraction was combined with 5x SDS-PAGE loading buffer, resolved by SDS-PAGE, transferred to a nitrocellulose membrane and immunoblotted with the indicated antibodies.

### Animal tissue experiments

Mouse hearts and kidneys were washed in ice-cold PBS, dounced in 2x SidBait lysis buffer and further lysed by sonication. The lysates were cleared by centrifugation at 21,300g for 20 minutes. Small molecule SidBait experiments in animal tissue lysates were performed as above.

### Yeast experiments

BY4741 strain *Saccharomyces cerevisiae* was grown in YDP media at 30 °C until confluency. Cells were pelleted at 3,000g for 10 minutes and resuspended in an equivalent volume of ice-cold SidBait lysis buffer (as detailed above). Yeast were lysed by vortexing using glass bead beating in flat bottom microcentrifuge tubes. Cleared cell lysates were obtained by two spins at 3,000g (2 min at 4 °C) and 20,000g (10 min at 4 °C). Lysates were then equilibrated to 5 mg/ml for subsequent SidBait experiments.

### *Xenopus* embryo experiments

Fertilized *Xenopus laevis* embryos were obtained by in vitro fertilization^56^ and were injected with 5 nL of bait (10 μM) followed immediately by 5 nL of SdeA^178−1100^ (5 mg/mL) at the one-cell stage and incubated for one hour at 14 °C. Two sets of embryos from different females were injected to obtain two biological replicates. Proteins were extracted in 1x MBS (Modified Barth Solution; 88 mM NaCl, 1 mM KCl, 2.4 mM NaHCO_3_, 15 mM HEPES pH 7.6, 0.3 mM CaNO_3_·4H_2_O, 0.41 mM CaCl_2_·6H_2_O, 0.82 mM MgSO_4_) containing 1% Triton X-100, Halt Protease phosphatase inhibitor (Thermo Fisher) and 5 mM EDTA. Yolk and debris were spun down at 16,000g at 4 °C for 30 min. The soluble fraction was incubated with 10 μl of streptavidin-magnetic beads (Pierce) for 1 h at RT. Beads were washed as above prior to mass spectrometry analysis.

### Intact mass spectrometry analysis

Protein samples were analyzed by LC–MS, using a Sciex X500B Q-TOF mass spectrometer coupled to an Agilent 1290 Infinity II HPLC. Samples were injected onto a POROS R1 reverse-phase column (2.1 mm × 30 mm, 20 μm particle size, 4,000 Å pore size) and desalted. The mobile phase flow rate was 300 μl min^−1^ and the gradient was as follows: 0–3 min, 0% B; 3–4 min, 0–15% B; 4–16 min, 15–55% B; 16–16.1 min, 55–80% B; 16.1–18 min, 80% B. The column was then re-equilibrated at the initial conditions before the subsequent injection. Buffer A contained 0.1% formic acid in water and buffer B contained 0.1% formic acid in acetonitrile.

The mass spectrometer was controlled by Sciex OS v.1.6.1 using the following settings: ion source gas 1, 30 psi; ion source gas 2, 30 psi; curtain gas, 35; CAD gas, 7; temperature, 300 °C; spray voltage, 5,500 V; declustering potential, 80 V; collision energy, 10 V. Data were acquired from 400–2,000 Da with a 0.5 s accumulation time and 4 time bins summed. The acquired mass spectra for the proteins of interest were deconvoluted using BioPharmaView v.3.0.1 (Sciex) to obtain the molecular mass values. The peak threshold was set to ≥5%, reconstruction processing was set to 20 iterations with a signal-to-noise threshold of ≥ 20 and a resolution of 2,500.

### On-bead digestion of SidBait samples and mass spectrometry analysis

Proteins on-beads were washed thoroughly prior to enzymatic digestion and LC-MS/MS analysis. Reduction and alkylation of cysteines was carried out first with 10 mM DTT (1 hr, 56 °C) and 50 mM iodoacetamine (45 min, RT in the dark), respectively. Proteins on-beads were digested overnight at 37°C with sequencing grade trypsin in 50 mM ammonium bicarbonate. The next day, tryptic peptides were acidified with 5% trifluoroacetic acid to stop digestion and desalted via solid phase extraction (SPE). LC-MS/MS experiments were performed on a Thermo Scientific EASY-nLC liquid chromatography system coupled to a Thermo Scientific Orbitrap Fusion Lumos mass spectrometer. To generate MS/MS spectra, MS1 spectra were first acquired in the Orbitrap mass analyzer (resolution 120,000). Peptide precursor ions were then isolated and fragmented using high-energy collision-induced dissociation (HCD). The resulting MS/MS fragmentation spectra were acquired in the ion trap. Label-free quantitative searches were performed using Proteome Discoverer 2.2 software (Thermo Scientific). Samples were searched against all entries included in the Human Uniprot database. Modifications included carbamidomethylation of cysteine (+57.021 Da), oxidation of methionine (+15.995 Da), and acetylation of peptide N-termini (+42.011 Da). Precursor and product ion mass tolerances were set to 10 ppm and 0.6 Da, respectively. Peptide spectral matches were adjusted to a 1% false discovery rate (FDR) and additionally proteins were filtered to a 5% FDR.

Protein abundances were quantified in SidBait-protein vs SidBait-empty samples by comparing area values of precursor ions. Abundance values were normalized across samples based on the total peptide amount identified in each. Samples with missing values were assigned a value equal to the average of the lower 1% of all abundance values. Enrichment scores were calculated as a ratio of the SidBait-protein:SidBait-empty abundances. Gene names and enrichment scores were plotted using Prism software.

### Crystallization, Data Collection and Structure Determination

CAMKIId^kd^ was prepared from *E. coli* as described above and diluted to 10 mg/mL in 10 mM Tris-HCl pH 8.0, 150 mM NaCl and 1 mM TCEP. CAMKIId^kd^:ribociclib was prepared by incubation of 350 μM CAMKIId^kd^ with 800 μM ribociclib for 1 hour. CAMKIId^kd^:ribociclib crystals were grown by the sitting drop vapor diffusion method overnight at 4 °C in 24-well Cryschem trays using a 1:1 ratio of protein/reservoir solution containing 0.2 M CaCl_2_, 20% w/v PEG 3,350. Growth of single crystals was initiated the following day by micro-seeding a Cryschem tray containing a 1:1 ratio of protein/reservoir solution containing 0.2 M CaCl_2_, 10% w/v PEG 3350. Wells were allowed to equilibrate for 24–48 hours and crystal growth was initiated by micro-seeding. CAMKIId^kd^:ribociclib crystals were cryo-protected with 0.2 M CaCl_2_, 0.15 M NaCl, 12% (w/v) PEG 3,350, 0.7 mM ribociclib and 35% (w/v) ethylene glycol, diffracted to a minimum Bragg spacing (*d*_min_) of 2.35 Å and exhibited the symmetry of space group P2_1_2_1_2_1_ with cell dimensions of a = 46.3 Å, b = 82.8 Å, c = 172.9 Å, and contained two CAMKIIδ^kd^:ribociclib per asymmetric unit. RavB^108-C^:CapZα:CapZβ^1−244^ was prepared by expression and purification from *E. coli* as described above and diluted to 10 mg/mL in 10 mM Tris-HCl pH 8.0, 150 mM NaCl and 1 mM TCEP. RavB:CapZ crystals were grown by the sitting drop vapor diffusion method at 20 °C in 24-well Cryschem trays using a 1:1 ratio of protein/reservoir solution containing 0.2 M ammonium formate, 19% w/v PEG 3,350, 20 mM 1,2-dioctanoyl-sn-glycero-3-(phosphoinositol-3-phosphate). Wells were allowed to equilibrate for 24–48 hours and crystal growth was initiated by micro-seeding. RavB:CapZ crystals were cryo-protected with 10 mM Tris pH 8.0, 0.2 M ammonium formate, 0.15 M NaCl, 21% w/v PEG 3,350 and 30% (w/v) ethylene glycol, diffracted to a minimum Bragg spacing (*d*_min_) of 2.00 Å and exhibited the symmetry of space group P2_1_ with cell dimensions of a = 66.5 Å, b = 55.3 Å, c = 77.9 Å, β = 107.1° and contained one RavB:CapZ per asymmetric unit. Diffraction data for CAMKIIδ^kd^:ribociclib were collected at beamline BL12–2 at the Stanford Synchrotron Radiation Lightsource (SLAC National Accelerator Laboratory, Menlo Park, California, USA). Diffraction data for RavB:CapZ crystals were collected on a Rigaku MicroMax-003 instrument outfitted with a copper sealed tube and a HyPix-6000HE direct photon detector. All diffraction data were processed in the program HKL-3000^57^ with applied corrections for effects resulting from absorption in a crystal and for radiation damage^58,59^, the calculation of an optimal error model, and corrections to compensate the phasing signal for a radiation-induced increase of non-isomorphism within the crystal^60,61^. Phases for CAMKIId^kd^:ribociclib were calculated via molecular replacement using residues 10–309 of PDB ID 3SOA^62^ as a search model. Phases for RavB:CapZ were calculated via molecular replacement using PDB ID 3AA7^63^ as a search model for the CapZα:CapZβ dimer. Molecular replacement for both structures was performed using the program PHASER^64^. A model for the RavB residues was automatically generated in the program Buccaneer^65^. Completion of models for both structures was performed by multiple cycles of manual rebuilding in the program Coot^66^ and refinement in the program Phenix^67^. Positional and isotropic atomic displacement parameter (ADP) as well as TLS ADP refinement was performed in the program Phenix with a random 7.0% of all data set aside for an *R*_*free*_ calculation. Data collection and structure refinement statistics are summarized in Table 1.

### Bacterial strains, cell lines, and culture media

*L. pneumophila* strains were grown in ACES-buffered yeast extract (AYE) broth or on ACES-buffered charcoal yeast extract (CYE) agar plates as previously described^68,69^. *E. coli* strains were grown in Luria-Bertani (LB) broth or on LB agar plates supplemented with 100 μg/mL ampicillin or 50 μg/mL kanamycin when appropriate.

*L. pneumophila* Philadelphia-1 wild-type (WT) strain Lp02 was a gift from Ralph Isberg. Lp02-mcherry was generated by expressing mCherry CDS cloned into pJB908^70^, a gift from Ralph Isberg.

COS-7, HeLa, and HEK293 cells were cultured in DMEM/High glucose with L-glutamine (ThermoFisher 11965092) supplemented with 10% FBS, 1% penicillin-streptomycin and incubated at 37 °C with 5% CO_2_.

H9 cells were cultured in mTeSR Plus according to standard protocols and were seeded in 6-well plates to start differentiation. Upon reaching 80–90% confluency, media was replaced with 3 μM CHIR99021 supplemented CDM3 (RPMI 1640; 0.5 mg/ml human albumin, ScienCell OsrHSA; 211 μg/ml L-ascorbic acid 2-phosphate) for 48 h. Subsequently, media was changed to 2 μM Wnt-C59 supplemented CDM3 for 48 hr. Then, media was changed every 2 days with CDM3 alone. On day 8 post-differentiation, samples were changed to RPMI 1640 + B27. Cells were analyzed for beating between Days 12–14 post differentiation. For SidBait experiments using cardiomyocytes, differentiations were performed as described with the following modification: on day 10 post-differentiation, 1 million cells were passaged onto 10 cm dishes coated in Matrigel and incubated with RPMI 1640 + B27 + 2 μM CHIR99021 to allow for expansion of cardiomyocytes.

### In vitro kinase activity assays

In vitro kinase assays using ribociclib were performed by Reaction Biology. IC_50_ values for ribociclib were measured against CaMKIId and CDK4/cyclinD1with a γ−^33^P radioactivity assay. Human CaMKIIδ was incubated with Ca^2+^-Calmodulin, (1 μM), a synthetic peptide substrate (KKLNRTLSFAEPG, 20 μM) and varying concentrations of ribociclib for 20 minutes at room temperature in base reaction buffer (20 mM HEPES pH 7.5, 10 mM MgCl_2_, 1 mM EGTA, 0.01% Brij35, 0.02 mg/ml BSA, 0.1 mM Na_3_VO_4_, 2 mM DTT, 1% DMSO). Human CDK4/cyclinD1 was incubated with Rb protein (3 μM) for 20 minutes at room temperature in base reaction buffer. Kinase reactions were initiated with 10 μM ^33^P-ATP and run for 2 hours. Phosphorylation of substrates was measured after blotting on P81 filter membranes.

### Ribociclib inhibition of human cardiomyocytes

Cardiomyocytes were differentiated and passaged as described above until 80% confluency and then treated with DMSO or indicated concentrations of ribociclib (Selleckchem S7440) for 1 hour and then stimulated with DMSO, 1 mM Ca^2+^, or 1 μM isoproterenol (Sigma-Aldrich I5752) to induce CaMKII activity for 30 minutes. Cells were lysed directly on the plate with 2x SDS-PAGE loading buffer, boiled, resolved by SDS-PAGE, transferred to a nitrocellulose membrane, and immunoblotted with the indicated antibodies.

### Heartbeat analysis

Cardiomyocytes were differentiated and passaged as described until 80% confluency and then stimulated with an additional 1 mM Ca^2+^ to induce CaMKII activity for 30 minutes and then treated with 1 μM ribociclib (Selleckchem S7440), 1 μM KN93 (Selleckchem S6787), or 1 μM flavipiridol (Selleckchem S1230) for one hour. Cells were visualised using a Zeiss Primovert Microscope and 4 × 30 second videos of each well were taken with an iPhone 11 rear-facing camera mounted directly to the right eyepiece.

### Isothermal titration calorimetry

Full length CapZ and RavB were purified as described above. For RavB^CPI^ measurements, a synthetic peptide consisting of RavB residues 108–148 (Genscript) was used. Protein and peptide samples were buffer exchanged into 50 mM Tris-HCl pH 8.0, 150 mM NaCl, 1 mM TCEP. All ITC experiments were performed on a Malvern MicroCal PEAQ ITC instrument. RavB^WT^ (100 μM), RavB^3R3A^ (100 μM) or RavB^CPI^ (300 μM) were injected into a cell containing CapZ (10 μM, 10 μM, or 30 μM, respectively) at 20 °C. The first injection was at a volume of 0.5 μL and followed by 20 injections of 1.9 μL. Data integration, fitting and error analysis were performed using NITPIC and SEDPHAT software. Results are reported as best fit with a 68.3% confidence interval. Figures were prepared using GUSSI software.

### Immunoprecipitation experiments

HEK293A cells were plated into a 6-well dish at 50% confluency. The following day, individual wells were transfected with pEGFP-C1, pEGFP-RavB^WT^, or pEGFP-RavB^3R3A^ using PolyJet (SignaGen SL100688) transfection reagent. Briefly, 1 μg plasmid DNA was added to 100 μL serum/antibiotic-free DMEM media and 3 μL PolyJet mixture, incubated at room temperature for 15 minutes, and added dropwise to the wells. The medium was replaced 5 hours after transfection (DMEM containing 10% FBS, 1% penicillin-streptomycin). After 24 h, cells were washed twice with ice cold PBS and lysed with 500 μL mammalian lysis buffer (50 mM Tris-HCl pH 8.0, 150 mM NaCl, 1 mM DTT, 1% Triton X-100, 1 mM ETDA, 1x cOmplete Protease Inhibitor Cocktail tablet). Lysates were cleared by centrifugation (4°C, 10 min, 21,300g), and 50 μL was saved for input. GFP-Catcher beads (Antibodies-online ABIN5311508) were washed twice in mammalian lysis buffer (4 °C, 2 min, 800g), and 20 μL packed beads per sample were added directly to the lysates and nutated overnight at 4 °C. The following day, beads were washed twice with mammalian lysis buffer (4 °C, 2 min, 800g), and conjugated proteins were eluted directly off the beads by boiling in 2x SDS-PAGE loading buffer for 10 mins. 40 μg of input and a fourth of total immunoprecipitated material were resolved by SDS-PAGE, transferred to a nitrocellulose membrane and immunoblotted with the indicated antibodies.

### Fluorescence microscopy

Fluorescence microscopy was performed as previously described^69^. Spinning disk confocal microscopy was performed with an Olympus IXplore SpinSR10 system (Olympus, Waltham, MA). Images were taken with a U Apo N TIRF 100x/1.49 oil objective (Olympus, Waltham, MA) and an Andor iXon Ultra 888 electron multiplier charge-coupled device (EM-CCD) camera (Oxford Instruments, Belfast, UK) when using the Olympus microscope.

For fluorescence imaging experiments, HeLa cells were seeded on an 8-well chamber coverglass at a density of ~2×10^4^ cells/well 1 day before transfection. The following day, individual wells were transfected with 35 ng of pEGFP-RavB^WT^, pEGFP-RavB3^R3A^, pEGFP-RavB^CTD^, or pEGFP-RavB^H214A^ and 100 ng of mTagBFP-Lamp1 (mTagBFP-Lysosomes-20 was a gift from Michael Davidson; Addgene plasmid #55263; RRID:Addgene_55263)^71^ using TransIT-X2 (mirusbio MIR 6000) transfection reagent per manufacturer’s instructions. Cells were fixed 18 hours after transfection.

For imaging *L. pneumophila* infection in mammalian cells, COS-7 cells expressing IgG FC receptor FcgRIIB was used as the model. COS-7 cells were seeded on collagen coated 8-well chamber coverglass at a density of ~2×10^4^ cells/well 1 day before transfection. The following day, individual wells were transfected with 35 ng of pEGFP-RavB^H214A^ or pEGFP-RavB^3R3A,H214A^ and 100 ng of pCCF- FcgRIIB using TransIT-X2 transfection reagent per manufacturer’s instructions. At the same time as transfection, *L. pneumophila-*mCherry strains were grown in AYE media. 16 hours post-transfection, stationary phase *L. pneumophila*-mCherry was opsonized with anti-*L. pneumophila* antibody (ThermoFisher AWB4CE4) in opti-MEM at 37 °C for 30 min, at the ratio of 10^6^ bacteria: 70 ng antibody: 50 μL of opti-MEM. Transfected COS-7 cells were washed once with PBS and infected with opsonized stationary phase *L. pneumophila-*mCherry at an MOI of 40. Infection was synchronized by centrifugation (RT, 5 min, 500g), incubated for another hour in normal culture conditions (37 °C, 5% CO_2_), then fixed.

Cells were washed once in PBS, then fixed in 4% paraformaldehyde (Electron Microscopy Sciences 15711 diluted in PBS) for 15 mins at room temperature (RT), washed with PBS, permeabilized with 0.1% Triton X-100 in PBS for 15 mins at RT, and blocked with 5% BSA in PBS for 10 minutes at RT. Indicated primary antibodies were incubated in 5% BSA in PBS at 1:400 dilution overnight at 4 °C. The following day, primary antibody was washed three times with PBS, followed by incubation with indicated Alexa fluorochrome conjugated secondary antibodies at 1:400 dilution in 5% BSA in PBS. Samples were washed three times in PBS and visualized by spinning disk confocal microscopy. All incubations were protected from light and agitated gently.

Colocalization statistics were computed by using Fiji/ImageJ built in Coloc 2 function. Regions of interest (ROI) were manually drawn using the polygon selections tool around the entirety of a single cell (excluding nucleus for RavB^H214A^ images). Coloc 2 (Single cell ROI, Threshold regression=Bisection, PSF=3.0, Costes randomizations=100) was run between the specified color channels, and the Pearson’s R value (above threshold) was noted and plotted in PRISM.

### Generation of rabbit polyclonal RavB antibody

His-tagged RavB was purified from Rosetta (DE3) *E. coli* by Ni-NTA affinity purification followed by size-exclusion chromatography. Proteins were sent to Cocalico Biologicals, Inc. for inoculation of rabbits. Anti-serum was received and the anti-RavB antibodies were partially purified by ammonium sulfate precipitation^72^. Briefly, 15 mL of the anti-serum was centrifuged (4 °C, 30 min, 10,000g), and incubated with 35 mL saturated ammonium sulfate (0.5 L 0.2 M sodium borate pH 8, 160 mM NaCl + 400 g (NH_4_)2SO_4,_ heated until dissolved, cooled at 4 °C overnight), with mixing for 2 h at 4°C. The mix was then spun (4 °C, 30 min, 10,000g) and supernatant discarded. The pellet was dissolved in 15 mL of BBS (0.2 M sodium borate pH 8, 160 mM NaCl) spun (4 °C, 10 min, 10,000g) and the supernatant collected.

RavB was purified as described above but using 50 mM HEPES instead of 50 mM Tris-HCl as buffer to omit primary amines. After size-exclusion chromatography, the protein was buffer exchanged into 0.2 M NaHCO_3_ pH 8.3, 0.5 M NaCl. A HiTrap NHS-activated High Performance column (Cytiva 17071701) was washed with 6 mL of 1 mM HCl, then RavB was injected and pumped back and forth for 30 mins. The column was then injected with 1 mL of 10 mM Tris pH 7.5, and deactivated with 6 mL Buffer A (0.5 M ethanolamine pH 8.3, 0.5 M NaCl) then 6 mL Buffer B (0.1 M acetic acid pH 4, 0.5 M NaCl), repeated three times for six total washes.

The protein-conjugated column was then washed with 10 mL of 10 mM Tris-HCl pH 7.5, then 10 mL of 100 mM glycine pH 2.5, 10 mM BBS. Fractionated serum was then loaded, and subsequently washed with 20 mL BBS, then 20 mL 10 mM Tris-HCl pH 7.5, 0.5 M NaCl. Finally, the antibody was eluted with 9 mL of 100 mM glycine pH 2.5 directly into a tube containing 1 M Tris 8.0. The antibody was concentrated using a centrifugal filter, sodium azide was added to final concentration of 0.02%, and the anti-RavB antibodies were stored at −20 °C until use.

### PIP strip lipid binding assay

PIP-strip (Echelon Biosciences P-6001) binding assay was conducted according to manufacturer’s instructions. All steps are performed with agitation at room temperature unless specified otherwise. Briefly, the membrane was blocked with 3% BSA in TBST (50 mM Tris-HCl pH 8.0, 150 mM NaCl, 0.1% Tween 20) for 1 hr. Purified RavB was diluted in 3% BSA to 1 μg/mL, and 5 mL was added to blocked membrane for 1 hr. The membrane was washed with TBST 3 times for 5 minutes each. Rabbit anti-RavB antibody was added at 1:1,000 dilution in 3% TBST, and incubated overnight at 4 °C. On the following day, the membrane was washed 3 times with TBST, and secondary anti-rabbit HRP antibody at 1:2,000 dilution in 3% TBST was added for 1 hr. This membrane was then washed 3 times with TBST, incubated with SuperSignal West Pico PLUS chemiluminescent substrate for 1 minute, dried, and exposed on autoradiography film.

### Liposome sedimentation assay

Liposomes containing POPC as carrier and various phospholipids were generated by mixing chloroform stocks of POPC and lipid (POPC, PI, PI3P, PI4P, PI5P, PI34P_2_, PI35P_2_, PI45P_2_, PIP_3_) (Echelon Biosciences) to form final concentration of 12.75 mM and 2.25 mM, respectively, in 1 mL volume. The lipids were then dried using a vacufuge, resuspended in 1 mL H O, and vortexed for 5 minutes. Liposomes were then subject to 5 freeze-thaw cycles with liquid nitrogen. Purified RavB protein was centrifuged (4 °C, 15 min, 21,300g) to exclude any aggregates. 2 μg protein was incubated with 112 μM lipid (1:20 dilution of liposome stock), in 20 μL of 50 mM Tris-HCl pH 8.0, 150 mM NaCl, 1 mM DTT for 1 hr at room temperature. Liposomes were sedimented by centrifugation (4 °C, 30 min, 21,300g). The supernatant was combined with 5x SDS-PAGE loading buffer, while the pellet was solubilized with 20 μL of 1x SDS-PAGE loading buffer. The samples were resolved by SDS-PAGE and stained with Coomassie to determine the presence of protein.

### Actin decapping and polymerization assays

Monomeric non-muscle actin (Cytoskeleton APHL99) was prepared following manufacturer’s instructions. Briefly, the desiccated protein was reconstituted by adding deionized water and pipetting on ice for 10 minutes. Actin was then diluted to 0.4 mg/ml in general actin buffer supplemented with ATP (5 mM Tris-HCl pH 8.0, 0.2 mM CaCl_2_, 0.2 mM ATP), and incubated on ice for 1 hour to depolymerize any oligomers. Any remaining oligomers were excluded by centrifugation (4 °C, 15 min, 21,300g), and the supernatant containing G-actin was collected.

Generation of short, uni-sized actin filaments decorated with capping protein^73^ was adapted from previous methods. Briefly, 500 μg actin monomers were incubated with molar excess (1,000 μg) purified chicken CapZ complex. After 2 mins at 4 °C, 10x ME buffer was added to yield 0.5 mM MgCl_2_, 0.2 mM EDTA final concentration for 1 min at 4 °C. Polymerization was initiated by addition of 10x KMEI buffer to yield 50 mM KCl, 1.5 mM MgCl_2_, 1 mM EGTA, 10 mM imidazole pH 7.0 final concentration for 2 min at room temperature. The protein solution was concentrated using a centrifugal filter until 500 μL final volume where it was separated by gel-filtration over a Superdex 200 increase 10/300 GL column in 1x KMEI buffer. 20 μL from each 500 μL fraction was collected, combined with 5x SDS-PAGE loading buffer, resolved by SDS-PAGE, and stained with Coomassie to determine protein-complex location.

Fractions containing actin-CapZ complex were combined, concentrated such that final Actin-CapZ-RavB solution was under 500 μL, and incubated with either molar excess (1,000 μg) purified RavB^WT^ or RavB^3R3A^ for 30 mins at 4 °C. The protein solution was then separated by gel filtration over a Superdex 200 increase 10/300 GL column in 1x KMEI buffer. 20 μL from each 500 μL fraction was collected, combined with 5x SDS-PAGE loading buffer, resolved by SDS-PAGE, and stained with Coomassie to determine protein-complex location.

For the actin polymerization assays, 2 μM G-actin-pyrene (prepared as by manufacturer’s instructions; Cytoskeleton BK003) was mixed with 50 nM CapZ and 1.25 μM of CBR115 or RavB^115–139^. For controls, an equal volume of buffer was added in place of CapZ or decapping peptides. Polymerization reactions were initiated by addition of 10x KMEI buffer (final volume: 60 μL) in a flat black 384-well plate (Greiner). After a 5 s shake, polymerization reactions were measured using a Tecan Spark Cyto plate reader (ex: 360 nm; em: 420 nm; 10 nm bandwidth) in 15 s intervals.

### Synthesis of linker


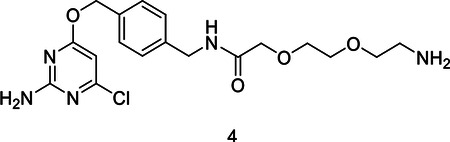


#### N-(4-(((2-amino-6-chloropyrimidin-4-yl)oxy)methyl)benzyl)-2-(2-(2-aminoethoxy)ethoxy)acetamide tert-butyl (2-(2-(2-((4-(((2-amino-6-chloropyrimidin-4-yl)oxy)methyl)benzyl)amino)-2-oxoethoxy)ethoxy)ethyl)carbamate.


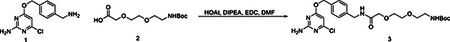


A solution of CLP-amine **1** (100.6 mg, 0.38 mmol) and 2,2-dimethyl-4-oxo-3,8,11-trioxa-5-azatridecan-13-oic acid **2** (119.5 mg, 0.45 mmol) in anhydrous DMF (1.9 ml) was cooled in an ice bath before the addition of 1-hydroxy-7-azabenzotriazole HOAt (71.0 mg, 0.52 mmol) and N,N-diisopropylethylamine DIPEA (198 μl, 1.1 mmol). The reaction was stirred cold for 5 minutes, after which N-(3-Dimethylaminopropyl)-N′-ethylcarbodiimide hydrochloride EDC (101.8 mg, 0.53 mmol) was added and the reaction was gently warmed to ambient temperature and stirred overnight. The reaction was diluted with EtOAc and washed with saturated NaHCO_3_ and brine. The organic layer was dried over Na_2_SO_4_, filtered and condensed. Crude reaction product was carried forward directly. ^1^H NMR (400 MHz, Chloroform-*d*) δ 7.36 (d, *J* = 8.1 Hz, 2H), 7.31 (d, *J* = 8.2 Hz, 2H), 7.22 (bs, 1H), 6.16 (s, 1H), 5.30 (d, *J* = 6.2 Hz, 4H), 5.14 (s, 2H), 4.71 (s, 1H), 4.49 (d, *J* = 5.9 Hz, 2H), 4.05 (s, 2H), 3.69 – 3.64 (m, 2H), 3.60 – 3.54 (m, 2H), 3.41 (t, *J* = 5.3 Hz, 2H), 3.14 (q, *J* = 5.4 Hz, 2H), 1.43 (s, 9H). ESI-MS (*m/z*): 510.1[M+H]^+^


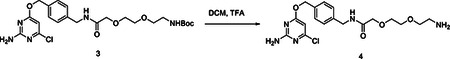


A solution of tert-butyl (2-(2-(2-((4-(((2-amino-6-chloropyrimidin-4-yl)oxy)methyl)benzyl)amino)-2-oxoethoxy)ethoxy)ethyl)carbamate **3** (156.3 mg, 0.31 mmol) in anhydrous DCM (2.5 ml) was cooled in an ice bath before the slow dropwise addition of trifluoroactic acid TFA (1.1 ml, 14.4 mmol). Lcms monitoring after 1 hour showed complete consumption of SM and desired m/z. The reaction was condensed and crude reaction mixture was carried forward. ^1^H NMR (400 MHz, Chloroform-*d*) δ 7.39 – 7.28 (m, 6H), 7.17 (dd, *J* = 7.9, 3.2 Hz, 1H), 6.16 (d, *J* = 0.9 Hz, 1H), 5.31 (s, 2H), 5.15 (s, 2H), 4.50 (d, *J* = 6.0 Hz, 3H), 4.06 (d, *J* = 2.1 Hz, 2H), 3.71 – 3.66 (m, 3H), 3.62 – 3.57 (m, 2H), 3.42 (t, *J* = 5.3 Hz, 2H), 2.73 (t, *J* = 5.3 Hz, 2H), 2.35 (s, 1H). ESI-MS (*m/z*): 410.1 [M+H]^+^


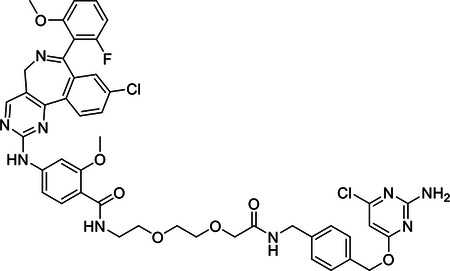


#### N-(2-(2-(2-((4-(((2-amino-6-chloropyrimidin-4-yl)oxy)methyl)benzyl)amino)-2-oxoethoxy)ethoxy)ethyl)-4-((9-chloro-7-(2-fluoro-6-methoxyphenyl)-5H-benzo[c]pyrimido[4,5-e]azepin-2-yl)amino)-2-methoxybenzamide


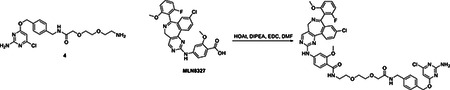


A solution of **4** (52.0 mg, 0.13 mmol) and MLN8237 (69.7 mg, 0.13 mmol) in anhydrous DMF (0.61 ml) was cooled in an ice bath before the addition of 1-hydroxy-7-azabenzotriazole (23.5 mg, 0.17 mmol) and N,N-diisopropylethylamine (64 μl, 0.37 mmol). The reaction was stirred cold for 5 minutes, after which N-(3-Dimethylaminopropyl)-N′-ethylcarbodiimide hydrochloride (34.8 mg, 0.18 mmol) was added and the reaction was gently warmed to ambient temperature and stirred overnight. Lcms monitoring indicated complete consumption of the amine and the desired m/z as the major product, along with a major and a minor byproduct. Both were less polar and smaller m/z than the desired. The reaction was diluted with EtOAc and washed with saturated NaHCO_3_ and brine. The organic layer with suspended solid was filtered. The isolated solid was mostly both by-products and about 20% of the desired. Most of the desired was in the filtrate of the organic layer, which was dried over Na_2_SO_4_, filtered and condensed. The crude mixture was purified by ISCO flash column chromatography in 0–15% MeOH/DCM to give 35.0 mg of pure product. Y=29 %. ^1^H NMR (400 MHz, Chloroform-*d*) δ 8.46 (s, 1H), 8.15 (d, *J* = 8.4 Hz, 1H), 8.08 (d, *J* = 8.6 Hz, 1H), 8.04 (t, *J* = 5.3 Hz, 1H), 7.96 (s, 1H), 7.85 (d, *J* = 2.0 Hz, 1H), 7.51 (dd, *J* = 8.4, 2.2 Hz, 1H), 7.40 (t, *J* = 6.0 Hz, 1H), 7.33 – 7.21 (m, 3H), 7.06 (d, *J* = 8.7 Hz, 1H), 6.65 (d, *J* = 59.5 Hz, 2H), 6.04 (s, 1H), 5.46 (s, 2H), 5.13 (s, 2H), 4.89 (d, *J* = 42.0 Hz, 1H), 4.40 (d, *J* = 6.0 Hz, 2H), 4.04 (s, 3H), 3.83 (s, 4H), 3.72 – 3.58 (m, 5H), 3.52 (dq, *J* = 14.3, 4.8 Hz, 5H). ESI-MS (*m/z*): 910.1 [M+H]^+^


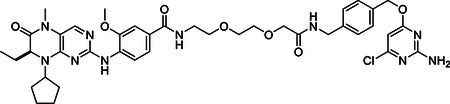


#### (S)-N-(2-(2-(2-((4-(((2-amino-6-chloropyrimidin-4-yl)oxy)methyl)benzyl)amino)-2-oxoethoxy)ethoxy)ethyl)-4-((8-cyclopentyl-7-ethyl-5-methyl-6-oxo-5,6,7,8-tetrahydropteridin-2-yl)amino)-3-methoxybenzamide


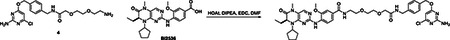


An emulsion of **4** (48.3 mg, 0.12 mmol) and BI2536 (55.5 mg, 0.13 mmol) in anhydrous DMF was cooled in an ice bath before the addition of 1-hydroxy-7-azabenzotriazole (21.4 mg, 0.16 mmol) and N,N-diisopropylethylamine (62 μl, 0.36 mmol). The reaction was stirred cold for 5 minutes, after which N-(3-Dimethylaminopropyl)-N′-ethylcarbodiimide hydrochloride (33.1 mg, 0.17 mmol) was added and the reaction was gently warmed to ambient temperature and stirred overnight in the absence of light. The reaction was diluted with EtOAc and washed with saturated NaHCO_3_ and brine. The organic layer was dried over Na_2_SO_4_, filtered and condensed. The crude mixture was purified by ISCO flash column chromatography in 0–10% MeOH/DCM to give 61.1 mg of pure product. Isolated yield=63 %. ^1^H NMR (400 MHz, Chloroform-*d*) δ 8.51 (d, *J* = 8.4 Hz, 1H), 7.65 (s, 1H), 7.58 (s, 1H), 7.39 (d, *J* = 1.9 Hz, 1H), 7.24 (q, *J* = 8.2 Hz, 6H), 6.56 (t, *J* = 5.5 Hz, 1H), 6.07 (s, 1H), 5.46 – 5.38 (m, 2H), 5.20 (s, 2H), 4.51 – 4.43 (m, 1H), 4.41 (d, *J* = 5.9 Hz, 2H), 4.19 (dd, *J* = 7.9, 3.7 Hz, 1H), 4.04 (s, 2H), 3.89 (s, 3H), 3.69 – 3.64 (m, 2H), 3.63 – 3.58 (m, 2H), 3.55 (t, *J* = 5.0 Hz, 2H), 3.49 (q, *J* = 6.0, 5.5 Hz, 2H), 3.43 (s, 1H), 3.29 (s, 3H), 2.18 – 2.04 (m, 2H), 1.97 (dq, *J* = 12.4, 8.7, 5.9 Hz, 1H), 1.88 – 1.73 (m, 4H), 1.73 – 1.59 (m, 4H), 0.84 (t, *J* = 7.5 Hz, 3H).). ESI-MS (*m/z*): 817.1 [M+H]^+^

## Supplementary Material

Supplement 1

Supplement 2

Supplement 3

Supplement 4

Supplement 5

Supplement 6

Supplement 7

Supplement 8

Supplement 9

1

## Figures and Tables

**Table 1.** Data collection and refinement statistics for CAMKIIδ^kd^:ribociclib and RavB:CapZ structures.

	CAMKIIδ^kd^:ribociclib	RavB:CapZ
**Data collection**		
Space group	P2_1_2_1_2_1_	P2_1_
Cell dimensions		
a, b, c (Å)	46.32, 82.82, 172.91	66.50, 55.32, 77.90
*α, β, γ* (°)	90, 90, 90	90, 107.08, 90
Wavelength (Å)	0.97946	1.5418
Resolution range (Å)	39.37 – 2.35 (2.39 – 2.35)	44.41 – 2.00 (2.03 – 2.00)
Unique reflections	28133 (1168)	35564 (1036)
Multiplicity	6.9 (4.9)	9.3 (1.3)
Completeness (%)	98.4 (82.6)	96.7 (56.6)
*R*_merge_ (%)	6.1 (79.1)	12.4 (35.9)
*R*_pim_ (%)	2.4 (35.7)	3.9 (34.5)
*I/σI*	28.9 (1.6)	19.3 (1.8)
CC_1/2_	0.74	0.79
Wilson B-value (Å^2^)	32.7	25.5
**Refinement**		
Resolution range (Å)	39.37 – 2.35 (2.41 – 2.35)	44.41 – 2.00 (2.04 – 2.00)
No. of reflections *R*_work_/*R*_free_	26175/1900 (823/67)	35046/2550 (769/61)
Data completeness (%)	91.7 (44.0)	95.21 (41.0)
Non-hydrogen atoms	4788	4585
protein	4641	4404
ligand	64	NA
solvent	73	181
*R*_*work*_	0.197 (0.251)	0.180 (0.263)
*R*_*free*_	0.251 (0.339)	0.208 (0.362)
R.m.s.d.		
Bond length (Å)	0.002	0.005
Bond angle (°)	0.558	0.712
Mean *B*-value		
chain A	41.2	31.6
chain B	52.3	27.0
chain C	NA	33.6
ligands	43.6	NA
solvent	33.5	28.4
Ramachandran favored (%)	96.9	97.2
Ramachandran allowed (%)	2.9	2.8
Ramachandran outliers (%)	0.2	0.0
Clashscore	2.1	1.5
Maximum likelihood coordinate error	0.29	0.20
Missing residues		
chain A	10, 302–309	1,2, 277–286
chain B	10, 297–309	NA
chain C	NA	109–114, 140–304

Values in parentheses are for highest resolution shell.

## References

[1] Isberg R. R., O’Connor T. J. & Heidtman M. The Legionella pneumophila replication vacuole: making a cosy niche inside host cells. Nat Rev Microbiol 7, 13–24 (2009). 10.1038/nrmicro1967 19011659 PMC2631402

[2] Bhogaraju S. Phosphoribosylation of Ubiquitin Promotes Serine Ubiquitination and Impairs Conventional Ubiquitination. Cell 167, 1636–1649.e1613 (2016). 10.1016/j.cell.2016.11.019 27912065

[3] Zhang M. Members of the Legionella pneumophila Sde family target tyrosine residues for phosphoribosyl-linked ubiquitination. RSC Chem Biol 2, 1509–1519 (2021). 10.1039/d1cb00088h 34704056 PMC8496037

[4] Qiu J. Ubiquitination independent of E1 and E2 enzymes by bacterial effectors. Nature 533, 120–124 (2016). 10.1038/nature17657 27049943 PMC4905768

[5] Zhang Q., Wan M., Kudryashova E., Kudryashov D. S. & Mao Y. Membrane-dependent actin polymerization mediated by the Legionella pneumophila effector protein MavH. PLoS Pathog 19, e1011512 (2023). 10.1371/journal.ppat.1011512 37463171 PMC10381072

[6] Bhogaraju S. Phosphoribosylation of Ubiquitin Promotes Serine Ubiquitination and Impairs Conventional Ubiquitination. Cell 167, 1636–1649 e1613 (2016). 10.1016/j.cell.2016.11.019 27912065

[7] Kotewicz K. M. A Single Legionella Effector Catalyzes a Multistep Ubiquitination Pathway to Rearrange Tubular Endoplasmic Reticulum for Replication. Cell Host Microbe 21, 169–181 (2017). 10.1016/j.chom.2016.12.007 28041930 PMC5300936

[8] Black M. H. Bacterial pseudokinase catalyzes protein polyglutamylation to inhibit the SidE-family ubiquitin ligases. Science 364, 787–792 (2019). 10.1126/science.aaw7446 31123136 PMC6767918

[9] Bhogaraju S. Inhibition of bacterial ubiquitin ligases by SidJ-calmodulin catalysed glutamylation. Nature 572, 382–386 (2019). 10.1038/s41586-019-1440-8 31330532 PMC6715450

[10] Sulpizio A. Protein polyglutamylation catalyzed by the bacterial calmodulin-dependent pseudokinase SidJ. eLife 8 (2019). 10.7554/eLife.51162 PMC6858067 31682223

[11] Gan N. Regulation of phosphoribosyl ubiquitination by a calmodulin-dependent glutamylase. Nature 572, 387–391 (2019). 10.1038/s41586-019-1439-1 31330531 PMC6855250

[12] Shin D. Regulation of Phosphoribosyl-Linked Serine Ubiquitination by Deubiquitinases DupA and DupB. Mol Cell 77, 164–179.e166 (2020). 10.1016/j.molcel.2019.10.019 31732457 PMC6941232

[13] Wan M. Deubiquitination of phosphoribosyl-ubiquitin conjugates by phosphodiesterase-domain-containing Legionella effectors. Proc Natl Acad Sci U S A 116, 23518–23526 (2019). 10.1073/pnas.1916287116 31690664 PMC6876201

[14] Kim S. & Isberg R. R. The Sde phosphoribosyl-linked ubiquitin transferases protect the Legionella pneumophila vacuole from degradation by the host. Proc Natl Acad Sci U S A 120, e2303942120 (2023). 10.1073/pnas.2303942120 37549300 PMC10437418

[15] Mackinnon A. L. & Taunton J. Target Identification by Diazirine Photo-Cross-linking and Click Chemistry. Curr Protoc Chem Biol 1, 55–73 (2009). 10.1002/9780470559277.ch090167 23667793 PMC3647699

[16] Roux K. J., Kim D. I., Raida M. & Burke B. A promiscuous biotin ligase fusion protein identifies proximal and interacting proteins in mammalian cells. J Cell Biol 196, 801–810 (2012). 10.1083/jcb.201112098 22412018 PMC3308701

[17] Rhee H. W. Proteomic mapping of mitochondria in living cells via spatially restricted enzymatic tagging. Science 339, 1328–1331 (2013). 10.1126/science.1230593 23371551 PMC3916822

[18] Tao A. J. A biotin targeting chimera (BioTAC) system to map small molecule interactomes in situ. Nat Commun 14, 8016 (2023). 10.1038/s41467-023-43507-5 38049406 PMC10695998

[19] O’Connor H. F. Ubiquitin-Activated Interaction Traps (UBAITs) identify E3 ligase binding partners. EMBO Rep 16, 1699–1712 (2015). 10.15252/embr.201540620 26508657 PMC4693525

[20] Hill Z. B., Pollock S. B., Zhuang M. & Wells J. A. Direct Proximity Tagging of Small Molecule Protein Targets Using an Engineered NEDD8 Ligase. J Am Chem Soc 138, 13123–13126 (2016). 10.1021/jacs.6b06828 27626304 PMC5308480

[21] Schenone M., Dancik V., Wagner B. K. & Clemons P. A. Target identification and mechanism of action in chemical biology and drug discovery. Nat Chem Biol 9, 232–240 (2013). 10.1038/nchembio.1199 23508189 PMC5543995

[22] Kalayil S. Insights into catalysis and function of phosphoribosyl-linked serine ubiquitination. Nature 557, 734–738 (2018). 10.1038/s41586-018-0145-8 29795347 PMC5980784

[23] Keppler A. A general method for the covalent labeling of fusion proteins with small molecules in vivo. Nat Biotechnol 21, 86–89 (2003). 10.1038/nbt765 12469133

[24] Bucko P. J. Subcellular drug targeting illuminates local kinase action. eLife 8 (2019). 10.7554/eLife.52220 PMC6930117 31872801

[25] Manfredi M. G. Characterization of Alisertib (MLN8237), an investigational small-molecule inhibitor of aurora A kinase using novel in vivo pharmacodynamic assays. Clinical cancer research : an official journal of the American Association for Cancer Research 17, 7614–7624 (2011). 10.1158/1078-0432.CCR-11-1536 22016509

[26] Adhikari B. PROTAC-mediated degradation reveals a non-catalytic function of AURORA-A kinase. Nat Chem Biol 16, 1179–1188 (2020). 10.1038/s41589-020-00652-y 32989298 PMC7610535

[27] Ciceri P. Dual kinase-bromodomain inhibitors for rationally designed polypharmacology. Nat Chem Biol 10, 305–312 (2014). 10.1038/nchembio.1471 24584101 PMC3998711

[28] Lombardo L. J. Discovery of N-(2-chloro-6-methyl- phenyl)-2-(6-(4-(2-hydroxyethyl)-piperazin-1-yl)-2-methylpyrimidin-4- ylamino)thiazole-5-carboxamide (BMS-354825), a dual Src/Abl kinase inhibitor with potent antitumor activity in preclinical assays. J Med Chem 47, 6658–6661 (2004). 10.1021/jm049486a 15615512

[29] Hortobagyi G. N. Overall Survival with Ribociclib plus Letrozole in Advanced Breast Cancer. N Engl J Med 386, 942–950 (2022). 10.1056/NEJMoa2114663 35263519

[30] Wu Y. Calmodulin kinase II is required for fight or flight sinoatrial node physiology. Proc Natl Acad Sci U S A 106, 5972–5977 (2009). 10.1073/pnas.0806422106 19276108 PMC2667018

[31] Chen P. Spectrum and Degree of CDK Drug Interactions Predicts Clinical Performance. Mol Cancer Ther 15, 2273–2281 (2016). 10.1158/1535-7163.MCT-16-0300 27496135

[32] Yang E. & Schulman H. Structural examination of autoregulation of multifunctional calcium/calmodulin-dependent protein kinase II. J Biol Chem 274, 26199–26208 (1999). 10.1074/jbc.274.37.26199 10473573

[33] Cho Y. S. 4-(Pyrazol-4-yl)-pyrimidines as selective inhibitors of cyclin-dependent kinase 4/6. J Med Chem 53, 7938–7957 (2010). 10.1021/jm100571n 21038853

[34] Lopez V. A. A Bacterial Effector Mimics a Host HSP90 Client to Undermine Immunity. Cell 179, 205–218 e221 (2019). 10.1016/j.cell.2019.08.020 31522888 PMC6754304

[35] Ho D. T., Bardwell A. J., Abdollahi M. & Bardwell L. A docking site in MKK4 mediates high affinity binding to JNK MAPKs and competes with similar docking sites in JNK substrates. J Biol Chem 278, 32662–32672 (2003). 10.1074/jbc.M304229200 12788955 PMC3017503

[36] Jumper J. Highly accurate protein structure prediction with AlphaFold. Nature 596, 583–589 (2021). 10.1038/s41586-021-03819-2 34265844 PMC8371605

[37] Edwards M. Capping protein regulators fine-tune actin assembly dynamics. Nat Rev Mol Cell Biol 15, 677–689 (2014). 10.1038/nrm3869 25207437 PMC4271544

[38] Nachmias N., Zusman T. & Segal G. Study of Legionella Effector Domains Revealed Novel and Prevalent Phosphatidylinositol 3-Phosphate Binding Domains. Infect Immun 87 (2019). 10.1128/iai.00153-19 PMC6529665 30962397

[39] Bruck S. Identification of a novel inhibitory actin-capping protein binding motif in CD2-associated protein. J Biol Chem 281, 19196–19203 (2006). 10.1074/jbc.M600166200 16707503 PMC2581424

[40] Hernandez-Valladares M. Structural characterization of a capping protein interaction motif defines a family of actin filament regulators. Nat Struct Mol Biol 17, 497–503 (2010). 10.1038/nsmb.1792 20357771 PMC3150215

[41] Chen T. T. Atypical Legionella GTPase effector hijacks host vesicular transport factor p115 to regulate host lipid droplet. Sci Adv 8, eadd7945 (2022). 10.1126/sciadv.add7945 PMC9757750 36525490

[42] Keeble A. H. Approaching infinite affinity through engineering of peptide-protein interaction. Proc Natl Acad Sci U S A 116, 26523–26533 (2019). 10.1073/pnas.1909653116 31822621 PMC6936558

[43] Guiley K. Z. p27 allosterically activates cyclin-dependent kinase 4 and antagonizes palbociclib inhibition. Science 366 (2019). 10.1126/science.aaw2106 PMC7592119 31831640

[44] Sumi N. J., Kuenzi B. M., Knezevic C. E., Remsing Rix L. L. & Rix U. Chemoproteomics Reveals Novel Protein and Lipid Kinase Targets of Clinical CDK4/6 Inhibitors in Lung Cancer. ACS Chem Biol 10, 2680–2686 (2015). 10.1021/acschembio.5b00368 26390342 PMC4684772

[45] Barber M. Cardiac arrhythmia considerations of hormone cancer therapies. Cardiovasc Res 115, 878–894 (2019). 10.1093/cvr/cvz020 30698686

[46] Bers D. M. & Grandi E. Calcium/calmodulin-dependent kinase II regulation of cardiac ion channels. J Cardiovasc Pharmacol 54, 180–187 (2009). 10.1097/FJC.0b013e3181a25078 19333131 PMC2784004

[47] Infante J. R. A Phase I Study of the Cyclin-Dependent Kinase 4/6 Inhibitor Ribociclib (LEE011) in Patients with Advanced Solid Tumors and Lymphomas. Clinical cancer research : an official journal of the American Association for Cancer Research 22, 5696–5705 (2016). 10.1158/1078-0432.CCR-16-1248 27542767 PMC5621377

[48] Klaeger S. The target landscape of clinical kinase drugs. Science 358 (2017). 10.1126/science.aan4368 PMC6542668 29191878

[49] Rashan E. H. ACAD10 and ACAD11 enable mammalian 4-hydroxy acid lipid catabolism. bioRxiv (2024). 10.1101/2024.01.09.574893 PMC12440821 40537578

[50] Trowbridge A. D. Small molecule photocatalysis enables drug target identification via energy transfer. Proc Natl Acad Sci U S A 119, e2208077119 (2022). 10.1073/pnas.2208077119 35969791 PMC9407219

[51] Kwak C. Identification of proteomic landscape of drug-binding proteins in live cells by proximity-dependent target ID. Cell Chem Biol 29, 1739–1753 e1736 (2022). 10.1016/j.chembiol.2022.10.001 36272407

[52] Montenegro R. C. Identification of molecular targets for the targeted treatment of gastric cancer using dasatinib. Oncotarget 11, 535–549 (2020). 10.18632/oncotarget.27462 32082487 PMC7007292

[53] Ozanne J., Prescott A. R. & Clark K. The clinically approved drugs dasatinib and bosutinib induce anti-inflammatory macrophages by inhibiting the salt-inducible kinases. Biochem J 465, 271–279 (2015). 10.1042/BJ20141165 25351958 PMC4286194

[54] Michard C. The Legionella Kinase LegK2 Targets the ARP2/3 Complex To Inhibit Actin Nucleation on Phagosomes and Allow Bacterial Evasion of the Late Endocytic Pathway. mBio 6, e00354–00315 (2015). 10.1128/mBio.00354-15 25944859 PMC4436068

[55] Wang D. Capping protein regulates endosomal trafficking by controlling F-actin density around endocytic vesicles and recruiting RAB5 effectors. Elife 10 (2021). 10.7554/eLife.65910 PMC8654373 34796874

[56] Sive H. L. & Harland R. M. Obtaining Xenopus Eggs and Embryos. Cold Spring Harb Protoc 2023, pdb top106195 (2023). 10.1101/pdb.top106195 36283836

[57] Minor W., Cymborowski M., Otwinowski Z. & Chruszcz M. HKL-3000: the integration of data reduction and structure solution--from diffraction images to an initial model in minutes. Acta Crystallogr D Biol Crystallogr 62, 859–866 (2006). 10.1107/S0907444906019949 16855301

[58] Borek D., Minor W. & Otwinowski Z. Measurement errors and their consequences in protein crystallography. Acta Crystallogr D Biol Crystallogr 59, 2031–2038 (2003). 14573959 10.1107/s0907444903020924

[59] Otwinowski Z., Borek D., Majewski W. & Minor W. Multiparametric scaling of diffraction intensities. Acta Crystallogr A 59, 228–234 (2003). 12714773 10.1107/s0108767303005488

[60] Borek D., Cymborowski M., Machius M., Minor W. & Otwinowski Z. Diffraction data analysis in the presence of radiation damage. Acta Crystallogr D Biol Crystallogr 66, 426–436 (2010). 10.1107/S0907444909040177 20382996 PMC2852307

[61] Borek D., Dauter Z. & Otwinowski Z. Identification of patterns in diffraction intensities affected by radiation exposure. J Synchrotron Radiat 20, 37–48 (2013). 10.1107/S0909049512048807 23254654 PMC3526920

[62] Chao L. H. A mechanism for tunable autoinhibition in the structure of a human Ca2+/calmodulin- dependent kinase II holoenzyme. Cell 146, 732–745 (2011). 10.1016/j.cell.2011.07.038 21884935 PMC3184253

[63] Takeda S. Two distinct mechanisms for actin capping protein regulation--steric and allosteric inhibition. PLoS Biol 8, e1000416 (2010). 10.1371/journal.pbio.1000416 20625546 PMC2897767

[64] McCoy A. J. Phaser crystallographic software. J Appl Crystallogr 40, 658–674 (2007). 10.1107/S0021889807021206 19461840 PMC2483472

[65] Cowtan K. The Buccaneer software for automated model building. 1. Tracing protein chains. Acta Crystallogr D Biol Crystallogr 62, 1002–1011 (2006). 10.1107/S0907444906022116 16929101

[66] Emsley P., Lohkamp B., Scott W. G. & Cowtan K. Features and development of Coot. Acta Crystallogr D Biol Crystallogr 66, 486–501 (2010). 10.1107/S0907444910007493 20383002 PMC2852313

[67] Adams P. D. PHENIX: a comprehensive Python-based system for macromolecular structure solution. Acta Crystallogr D Biol Crystallogr 66, 213–221 (2010). 10.1107/S0907444909052925 20124702 PMC2815670

[68] Chatfield C. H. & Cianciotto N. P. Culturing, media, and handling of *Legionella*. Methods in Molecular Biology 954, 151–162 (2013). 10.1007/978-1-62703-161-5_7 23150393

[69] Hsieh T. S. Dynamic remodeling of host membranes by self-organizing bacterial effectors. Science 372, 935–941 (2021). 10.1126/science.aay8118 33927055 PMC8543759

[70] Sexton J. A., Yeo H. J. & Vogel J. P. Genetic analysis of the *Legionella pneumophila* DotB ATPase reveals a role in type IV secretion system protein export. Molecular Microbiology 57, 70–84 (2005). 10.1111/j.1365-2958.2005.04667.x 15948950

[71] Rizzo M. A., Davidson M. W. & Piston D. W. Fluorescent protein tracking and detection: fluorescent protein structure and color variants. Cold Spring Harb Protoc 2009, pdb top63 (2009). 10.1101/pdb.top63 20150100

[72] Kent U. M. Purification of antibodies using ammonium sulfate fractionation or gel filtration. Methods Mol Biol 115, 11–18 (1999). 10.1385/1-59259-213-9:11 10098159

[73] Funk J. A barbed end interference mechanism reveals how capping protein promotes nucleation in branched actin networks. Nat Commun 12, 5329 (2021). 10.1038/s41467-021-25682-5 34504078 PMC8429771

