# Supplementary material for: Bacterial ubiquitin ligase engineered for small molecule and protein target identification
Source: bioRxiv. 2025 Mar 22:2025.03.20.644192. Preprint. [Version 1] doi: 10.1101/2025.03.20.644192 (PMC11957136; doi:10.1101/2025.03.20.644192)
Supplement: 1 [file NIHPP2025.03.20.644192V1-supplement-1.pdf]

bioRxiv preprint doi: https://doi.org/10.1101/2025.03.20.644192; this version posted March 22, 2025. The copyright holder for this preprint (which was not certified by peer review) is the author/funder, who has granted bioRxiv a license to display the preprint in perpetuity. It is made available under aCC-BY 4.0 International license.

Supplementary Figures

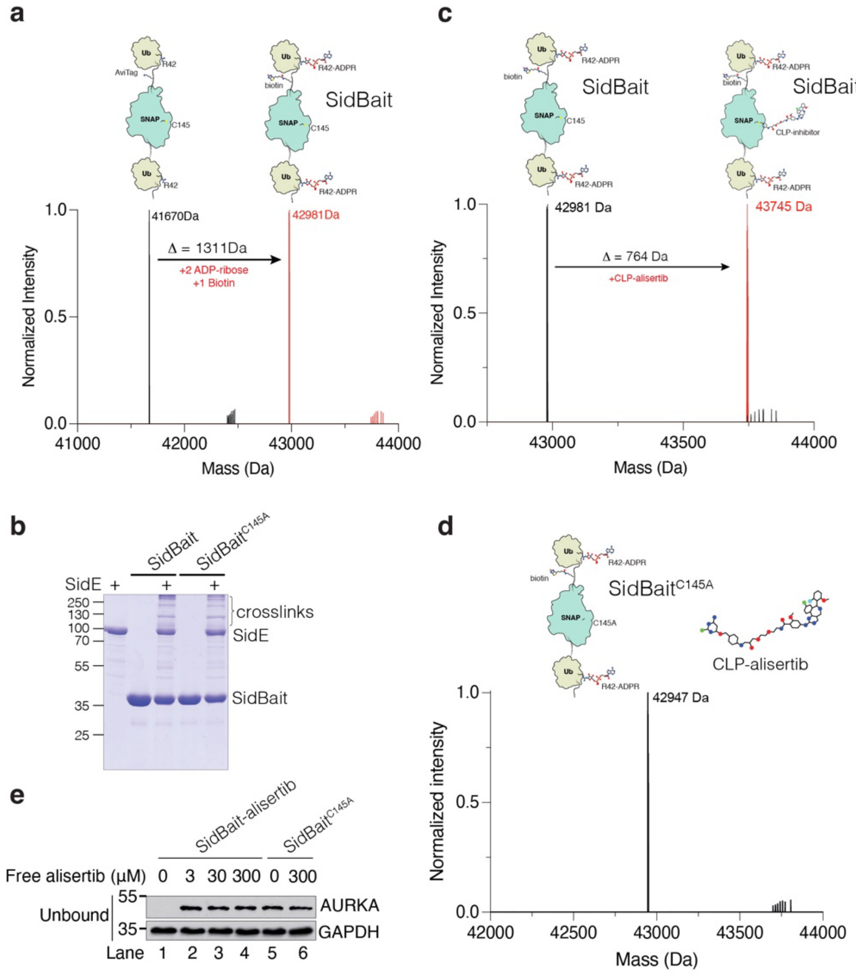

Supplementary Figure 1: SidBait identifies targets of small molecules.

(a) Intact mass spectrum of unmodified SidBait (left, black) and SidBait which contains a biotin and two ADP-ribose molecules (right, red).

(b) NAD^+-independent SidE autoubiquitination of the SidBait probe. The SidBait probe was incubated with $SidE^{PDE}$ and the reaction products were separated by SDS-PAGE and visualized by Coomassie staining. Crosslinking of the $SidBait^{C145A}$ control (right), which cannot conjugate CLP-derivatives of small molecules, demonstrates an otherwise functional SidBait construct.

(c) Intact mass spectrum of SidBait (left, black) and SidBait-alisertib (right, red).

(d) Protein immunoblotting of the unbound fractions following avidin enrichment of the SidBait-alisertib probe from HEK293 cell lysates that have been incubated with and without free alisertib. AURKA and GAPDH are shown. All cellular AURKA is bound to the SidBait-alisertib probe in the absence of free alisertib (lane 1). Following the addition of free alisertib, the SidBait-alisertib probe is competed off AURKA (lanes 2-4).

(e) Intact mass spectrum of the $SidBait^{C145A}$ control after incubation with CLP-alisertib, showing that the mutant protein cannot incorporate the small molecule.

bioRxiv preprint doi: https://doi.org/10.1101/2025.03.20.644192; this version posted March 22, 2025. The copyright holder for this preprint (which was not certified by peer review) is the author/funder, who has granted bioRxiv a license to display the preprint in perpetuity. It is made available under aCC-BY 4.0 International license.

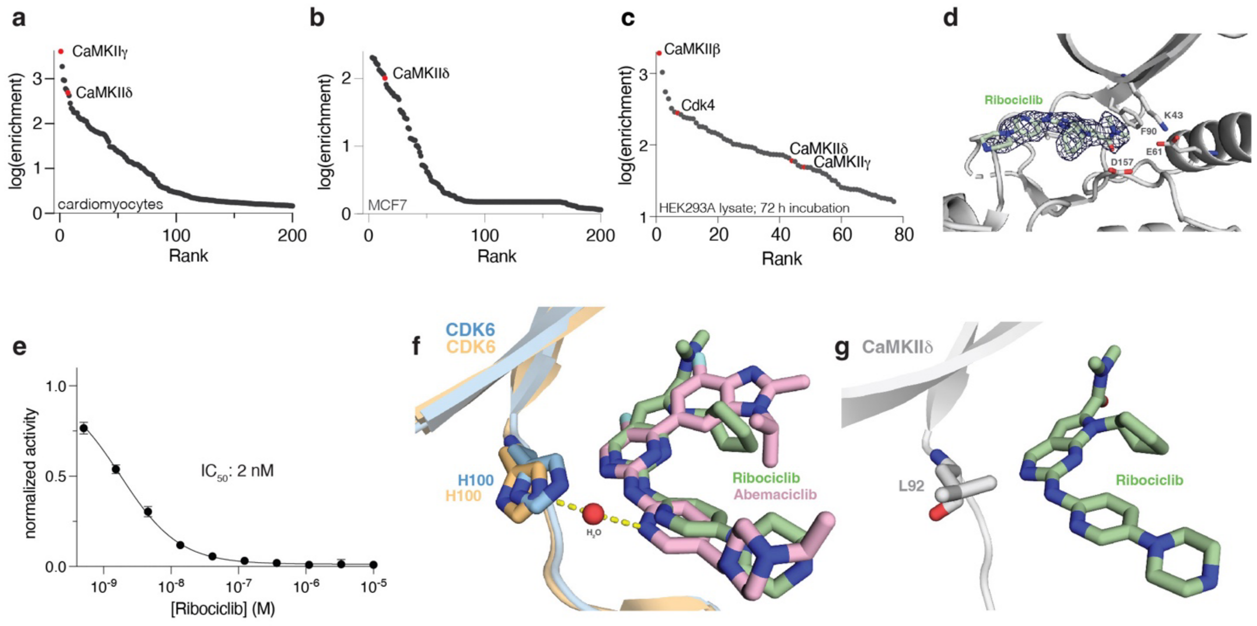

Supplementary Figure 2: SidBait identifies CaMKII as a target of the CDK4/6 inhibitor ribociclib.

(a, b) Plot of the fold enrichment of proteins from SidBait-ribociclib experiments in cultured cardiomyocytes **(a)** and MCF7 cells **(b)**.

(c) Plot of the fold enrichment of proteins from SidBait-ribociclib experiments in HEK293A cells following a 72-hour incubation with the bait

(d) A view of ribociclib in the active site of the CaMKII kinase domain. The $2F_o$-F_c electron density map, contoured to 1s, is represented by a dark blue mesh.

(e) In vitro CDK4/cyclinD1 activity assay in the presence of varying concentrations of ribociclib. The IC_{50} of ribociclib is shown in the inset. Error bars represent the S.E.M. of three replicates.

(f, g) Structural comparison between CDK6 and CAMKII bound to inhibitors. Structures of ribociclib and abemaciclib in the active site of CDK6 **(f)**, showing bridging interaction through an ordered water molecule. Ribociclib in CaMKII **(g)** does not exhibit this interaction.

bioRxiv preprint doi: https://doi.org/10.1101/2025.03.20.644192; this version posted March 22, 2025. The copyright holder for this preprint (which was not certified by peer review) is the author/funder, who has granted bioRxiv a license to display the preprint in perpetuity. It is made available under aCC-BY 4.0 International license.

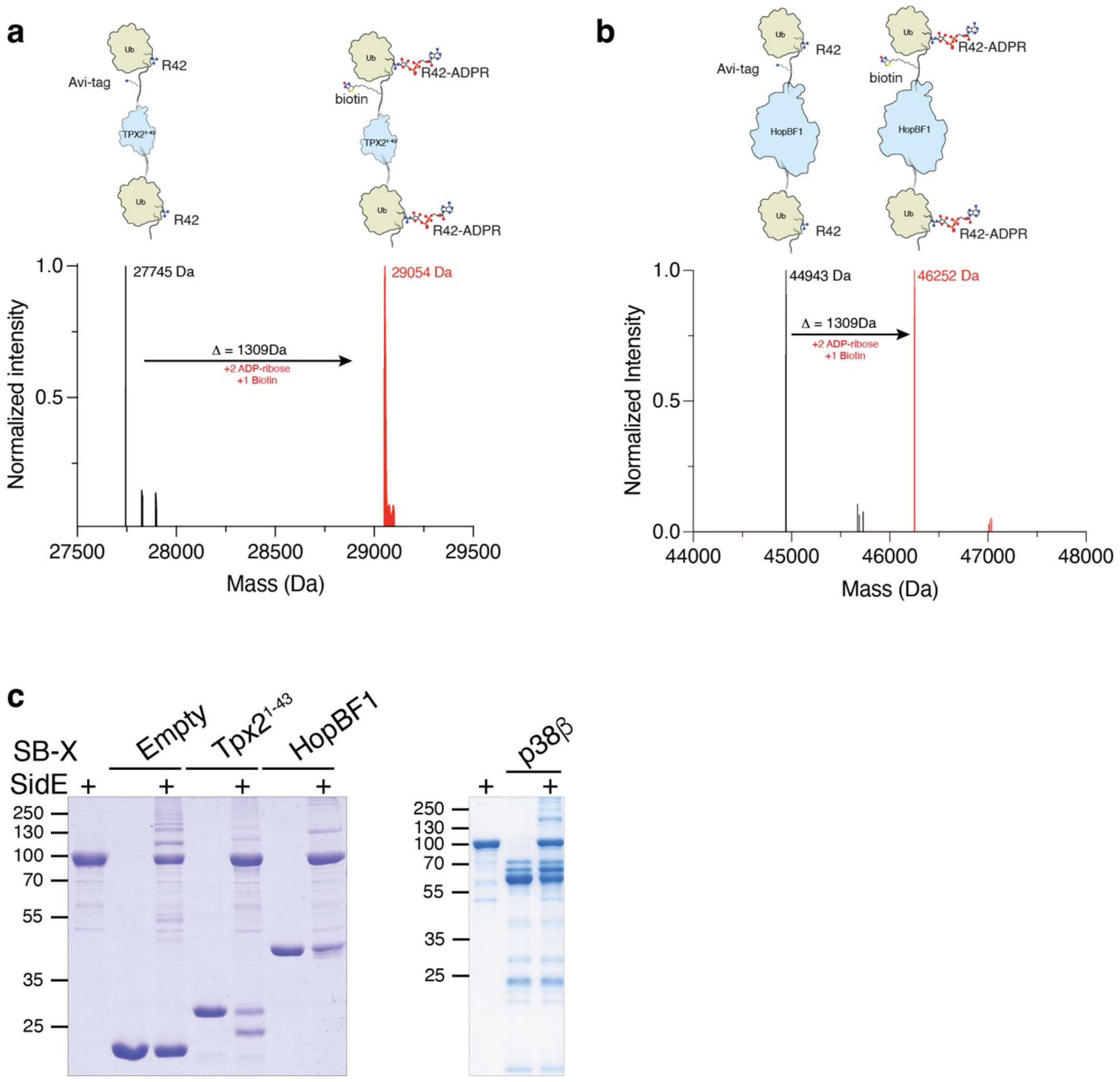

Supplementary Figure 3: SidBait identifies targets of proteins of interest.

(a, b) Intact mass spectra of unmodified SidBait-Tpx2$^{1-43}$ **(a)** and SidBait-HopBF1 **(b)** (left, black), and the respective SidBait-POI molecules containing a biotin and two ADP-ribose molecules (right, red).

(c) NAD^{+}-independent SidE autoubiquitination of the SidBait-POI probes. The probes were incubated with SidE and the reaction products were separated by SDS-PAGE and visualized by Coomassie staining.

bioRxiv preprint doi: https://doi.org/10.1101/2025.03.20.644192; this version posted March 22, 2025. The copyright holder for this preprint (which was not certified by peer review) is the author/funder, who has granted bioRxiv a license to display the preprint in perpetuity. It is made available under aCC-BY 4.0 International license.

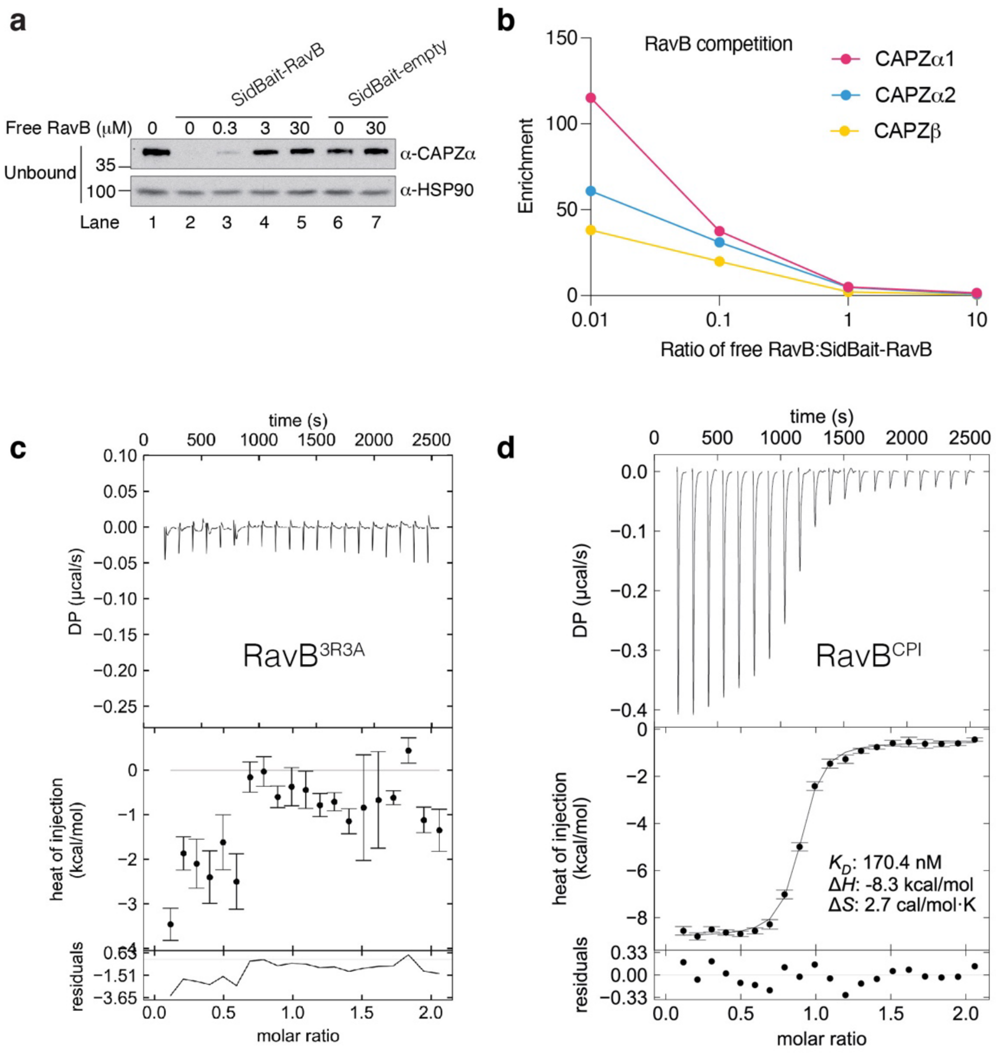

Supplementary Figure 4: SidBait identifies CapZ as a binding partner of the *Legionella* effector RavB.

(a) Protein immunoblotting of the unbound fractions following avidin enrichment of the SidBait-RavB probe from HEK293 cell lysates that have been incubated with and without free RavB. CapZα and HSP90 are shown. CapZ is readily detectable in a cell lysate (lane 1). All cellular CapZ is bound to the SidBait-RavB probe in the absence of free RavB (lane 2). Following the addition of free RavB, the SidBait-RavB probe is competed off RavB (lanes 3-5).

(b) Plots of the decreasing fold enrichment of CapZ isoforms from SidBait-RavB probe as quantified by mass spectrometry after addition of increasing competing free RavB.

(c, d) Isothermal titration calorimetry (ITC) traces showing the binding of RavB^{3R3A} **(c)** or $\text{RavB}^{108\text{-}148}$ containing the RavB^{CPI} **(d)** to CapZ. In each ITC experiment, the RavB species was injected into the cell containing CapZ. K_d, enthalpy and entropy values are shown in the inset. These values are undefined for RavB^{3R3A}, as no binding was observed.

bioRxiv preprint doi: https://doi.org/10.1101/2025.03.20.644192; this version posted March 22, 2025. The copyright holder for this preprint (which was not certified by peer review) is the author/funder, who has granted bioRxiv a license to display the preprint in perpetuity. It is made available under aCC-BY 4.0 International license.

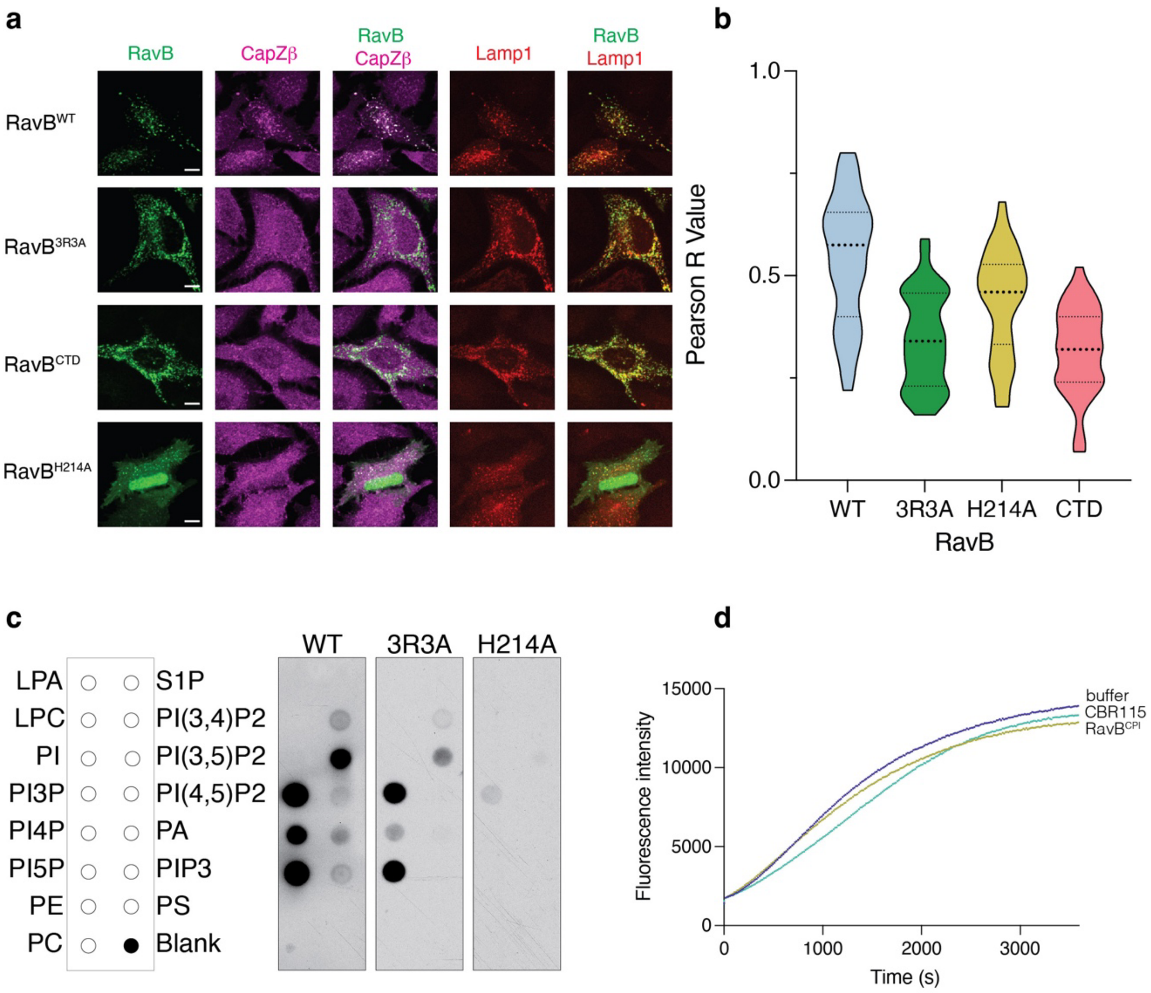

Supplementary Figure 5: RavB is a phosphatidylinositol phosphate binding, actin decapping protein.

(a) Immunofluorescence microscopy of HeLa cells expressing EGFP-RavB^{WT} or various mutants, mTagBFP-Lamp1. Endogenous CapZβ is also shown (magenta). The images depict the entire cell from **Figure 5a**. Scale bar represents 10 μm.

(b) Plot of the Pearson R Value of colocalization between transfected mTagBFP-Lamp1 and endogenous CapZβ in cells expressing WT or various RavB mutants. Each comparison was calculated using 40 cells across 3 independent experiments.

(c) Protein immunoblotting of RavB or mutants bound to a lipid panel spotted on PIP strips with an anti-RavB antibody. All membranes shown were exposed on the same film.

(d) Pyrene-actin polymerization assays demonstrating that the RavB^{CPI} does not affect actin polymerization. The polymerization of actin was measured in the presence of a buffer control (blue), the known decapping peptide CBR115 (teal) and the RavB^{CPI} peptide (green). Assays were run with 2 μM G-actin and 1.25 μM decapping peptide.

bioRxiv preprint doi: https://doi.org/10.1101/2025.03.20.644192; this version posted March 22, 2025. The copyright holder for this preprint (which was not certified by peer review) is the author/funder, who has granted bioRxiv a license to display the preprint in perpetuity. It is made available under aCC-BY 4.0 International license.

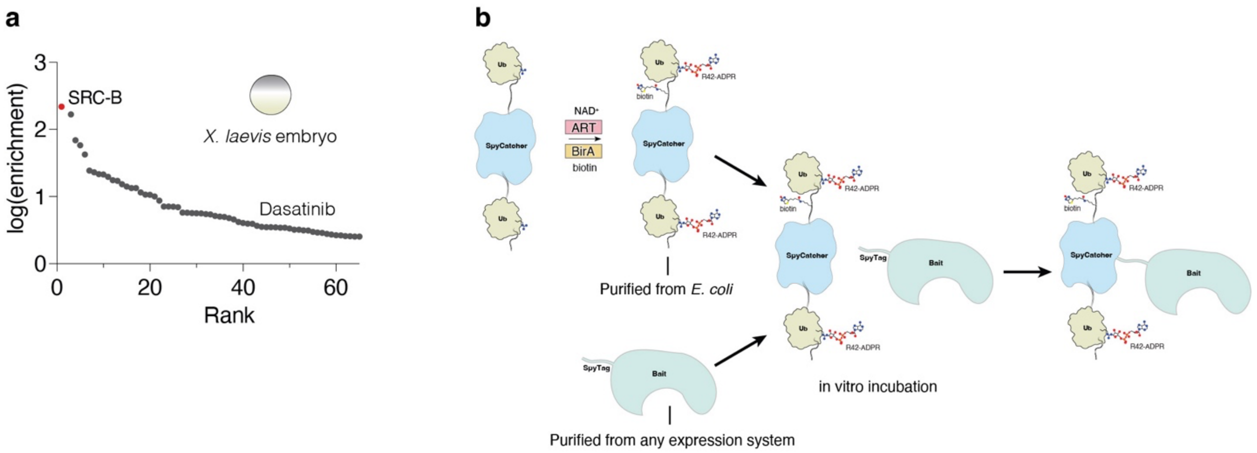

Supplementary Figure 6: Additional applications of SidBait.

(a) Plots of fold enrichment of proteins from SidBait-dasatinib in live *X. laevis* embryos over pulldowns with the SidBait^{C145A} control

(b) Schematic of the SpyTag/SpyCatcher system as applied to SidBait. The Ub-SpyCatcher-Ub fusion protein is coexpressed in *E. coli* with the SidE ART domain and BirA. The ADP-ribosylated and biotinylated protein is incubated with a protein of interest (POI) fused to the SpyTag, which has been purified from an alternative expression system. Upon mixing, the SpyTag-POI spontaneously conjugates to the SpyCatcher protein, forming a stable isopeptide bond. The resulting fusion protein is used in a SidBait experiment.
